# Maintaining essential health services during COVID-19: cross-country lessons of health system resilience from Asia, Sub-Saharan Africa and Latin America

**DOI:** 10.1136/bmjgh-2023-013392

**Published:** 2025-10-13

**Authors:** Moytrayee Guha, Arielle Cohen Tanugi-Carresse, Lucia Mullen, Sara Bennett, Rhoda Kitti Wanyenze, Andrea M Prado, Piya Hanvoravongchai, Magdalena Rathe, Julius Fobil, Ravindra Prasan Rannan-Eliya, Andy A Pearson, Claudio A Mora-García, Paul Cheh, Rawlance Ndejjo, Steven Ndugwa Kabwama, Duah Dwomoh, Suzanne Kiwanuka, Wasin Laohavinij, Melanie Coates, Laura Rathe, Nilmini Wijemunige, Zachary Hennenfent, William Wang, Siobhan Lazenby, Anne Liu, Jennifer B Nuzzo

**Affiliations:** 1 Brown University School of Public Health, Providence, Rhode Island, USA; 2 Health, Nutrition, and Population Global Practice, World Bank, Washington, DC, USA; 3 Department of International Health, Johns Hopkins University Bloomberg School of Public Health, Baltimore, Maryland, USA; 4 Johns Hopkins Center for Health Security, Baltimore, Maryland, USA; 5 Department of Disease Control and Environmental Health, School of Public Health, Makerere University, Kampala, Uganda; 6 INCAE Business School, Alajuela, Costa Rica; 7 Thailand National Health Foundation, Bangkok, Thailand; 8 Faculty of Medicine, Chulalongkorn University, Bangkok, Thailand; 9 Fundacion Plenitud, Santo Domingo, Dominican Republic; 10 School of Public Health, University of Ghana, Accra, Ghana; 11 Institute for Health Policy, Colombo, Sri Lanka; 12 World Health Organization Regional Office for the Western Pacific, Manila, Philippines; 13 Department of Community Health and Behavioural Sciences, School of Public Health, Makerere University, Kampala, Uganda; 14 Department of Health Policy Planning and Management, School of Public Health, Makerere University, Kampala, Uganda; 15 Global Health Partnerships, Health Education England, London, UK; 16 Gates Ventures LLC, Seattle, Washington, USA; 17 London School of Hygiene & Tropical Medicine, London, UK; 18 Pandemic Center, Brown University School of Public Health, Providence, Rhode Island, USA; 19 Department of Epidemiology, Brown University School of Public Health, Providence, Rhode Island, USA

**Keywords:** COVID-19, Health systems, Control strategies, Public Health, Health services research

## Abstract

The COVID-19 pandemic severely disrupted the delivery of essential health services (EHS) worldwide, contributing to excess morbidity and mortality from preventable conditions. Some countries employed innovative strategies that may have enabled their health systems to be more resilient than others in responding to COVID-19. This cross-country analysis aimed to identify beneficial practices and policies employed by six low- and middle-income countries (LMICs) in Asia, Sub-Saharan Africa and Latin America to maintain access to EHS while responding to COVID-19. Cross-country research partners (CCRPs) led a mixed methods assessment to identify best practices and strategies for COVID-19 response and continued provision of EHS between April 2021 and September 2022. A cross-country analysis was conducted to extract and thematically code best practices that were reported as beneficial by three or more study countries based on desk reviews, key informant interviews and quantitative and qualitative analyses. Cross-cutting enablers, barriers and lessons learnt were also documented. Cross-country themes include whole-of-government approaches; multisectoral collaboration and decision-making; early outbreak control measures; partnerships with the private sector; innovations in service delivery and health financing; a robust health workforce; adaptation of existing disease response capacities; and community engagement. Long-standing investments in health systems strengthening and preparedness, integrated health systems, public trust in government, leadership and political will, prior experience in responding to epidemics, strong primary healthcare systems, existing health financing mechanisms and provision of social and economic supports were identified as cross-cutting enablers. Lack of context-specific definitions for EHS, inequitable access to technology and lack of access to real-time, high-quality data were identified as challenges in study countries. This study provides valuable insights into the practices that may be considered beneficial and worthy of pursuit by other countries wishing to strengthen health system resilience and preparedness for future health emergencies. Further research is needed to evaluate the effectiveness of these practices in different settings.

**SUMMARY BOX**

- Weak and unprepared health systems struggled to respond to the health and economic shocks of the COVID-19 pandemic. Studies have documented concerning declines in access to and utilisation of essential health services (EHS), jeopardising decades of progress, particularly in LMICs. Some countries have also experienced declines in life expectancy.
- The pandemic highlighted the need for stronger health systems that are prepared for future public health emergencies, but effective strategies are not well understood.
- Despite growing literature on health system resilience, there is a lack of focus on operational strategies and best practices to strengthen resilience in resource-limited settings.
- While there has been extensive coverage of countries’ COVID-19 response efforts, few reports have catalogued beneficial practices employed by LMICs to maintain access to EHS while responding to the pandemic. This study aims to fill this knowledge gap, providing insights into interventions that enabled resilience and continued provision of EHS in resource-limited settings.
- This analysis is among the first to document cross-country practices, operational strategies and lessons learnt from diverse regions in Asia, Africa and Latin America. Cross-cutting themes include whole-of-government approaches, multisectoral collaboration and decision-making, partnerships with the private sector and academia, innovations in service delivery and health financing, a robust health workforce and community engagement.
- This practice paper synthesises key insights and actionable solutions for strengthening health system resilience and preparedness for health emergencies in comparable settings.

## Introduction

 COVID-19 exposed the devastating consequences of underinvestment in health systems. Weak and unprepared health systems struggled to respond to the unprecedented health and economic shocks arising from the pandemic, leading to widespread direct and indirect effects on population health. Across the globe, several countries reported historic declines in life expectancy, in part due to the loss of life from COVID-19, but also as a result of the disruption to healthcare services.^1 2^ As health systems focused their efforts on responding to COVID-19 and diverted human, financial and material resources away from existing programmes, the provision of routine and preventative health services has suffered. In a 2020 survey of 105 countries, the WHO found that 90% of countries reported prolonged disruptions to essential health services (EHS) since the start of the COVID-19 pandemic.^3^ Even high-performing and well-resourced health systems have faced challenges in maintaining access to EHS. Maternal, child and reproductive health, immunisation, HIV/AIDS, TB and non-communicable disease (NCD) services have been most commonly disrupted, resulting in excess mortality and morbidity from preventable causes.^12 4^,^8^

Recent studies have documented concerning declines in access to and utilisation of EHS, jeopardising decades of progress in improving health outcomes, especially in low- and middle-income countries (LMICs).^19^,^11^ Several reasons have been cited for declining healthcare use during the pandemic, including the public’s fear of becoming infected while visiting health facilities, the suspension or cancellation of non-COVID-19 care as well as barriers imposed by lockdown policies.^1 2^ Although some services can potentially be delayed during an emergency, other, more urgent, services cannot.

The pandemic has revealed the urgent need for countries to strengthen health systems and equip them to be resilient to future health emergencies.^612^,^15^ Health system resilience encompasses the capacity to anticipate, absorb, respond and recover from shocks, while maintaining core functions and serving the ongoing and acute care needs of communities.^2^ Despite unprecedented challenges posed by the pandemic, some countries have employed innovative strategies that may have allowed their health systems to be more resilient than others in responding to COVID-19 and maintaining access to routine preventative and therapeutic EHS.

We sought to better understand what national approaches promote health systems resilience in LMICs. This research study identified six countries in Asia, Sub-Saharan Africa and Latin America that demonstrated evidence of having been able to maintain EHS while responding to pandemic-related surges in demand for healthcare and maintaining COVID-19 prevention and control measures. For each of the six countries, we identified national practices and policies that were likely beneficial in allowing each country to respond to the pandemic and provide medical care during surges of COVID-19 cases, while also providing EHS. A comprehensive analysis of each country’s efforts to continue EHS and enhance health system resilience was conducted to identify noteworthy policies, practices and lessons learnt.

This research study is part of the Exemplars in Global Health (EGH) initiative based at Gates Ventures. The EGH initiative brings together experts, funders and local partners with the mission of identifying positive global health outliers, analysing what makes countries successful and disseminating core lessons that can be replicated in comparable settings. A subset of the EGH initiative focuses on best practices and lessons learnt in maintaining EHS during large-scale public health emergencies. Launched in 2020, the Exemplars in COVID-19 Response (ECR) initiative aims to identify countries that excelled in mitigating the impact of the pandemic on EHS delivery. This study seeks to understand the impact of the COVID-19 pandemic on the provision of EHS and health system resilience.

While there has been extensive coverage of countries' COVID-19 response efforts, few reports have catalogued the strategies and best practices of LMICs that maintained EHS while responding to COVID-19. This analysis is among the first to document cross-country practices, operational strategies and lessons learnt from diverse regions in Asia, Africa and Latin America. This study aims to fill this knowledge gap, providing insights into policies and practices that enabled resilience and the continued provision of EHS in low-resource settings during the pandemic. The cross-country findings offer beneficial practices and actionable solutions to strengthen health system resilience and preparedness for future public health emergencies.

### Selection of countries and research partners to conduct deep-dive research

Cross-country research partners (CCRPs) from Brown University School of Public Health, Johns Hopkins Bloomberg School of Public Health (JHSPH) and Makerere University SPH (MakSPH) led the ECR research consortium and oversaw a mixed methods assessment to identify beneficial policies and practices for COVID-19 response and maintenance of EHS across six LMICs between April 2021 and September 2022. Prior to initiating this research, each of the CCRPs sought ethical clearance from their respective institutions. In-country research partners (ICRPs) followed country procedures to obtain ethical clearance for the project. The Institutional Review Board of JHSPH exempted the cross-country analysis part of the study.

**Figure 1:**
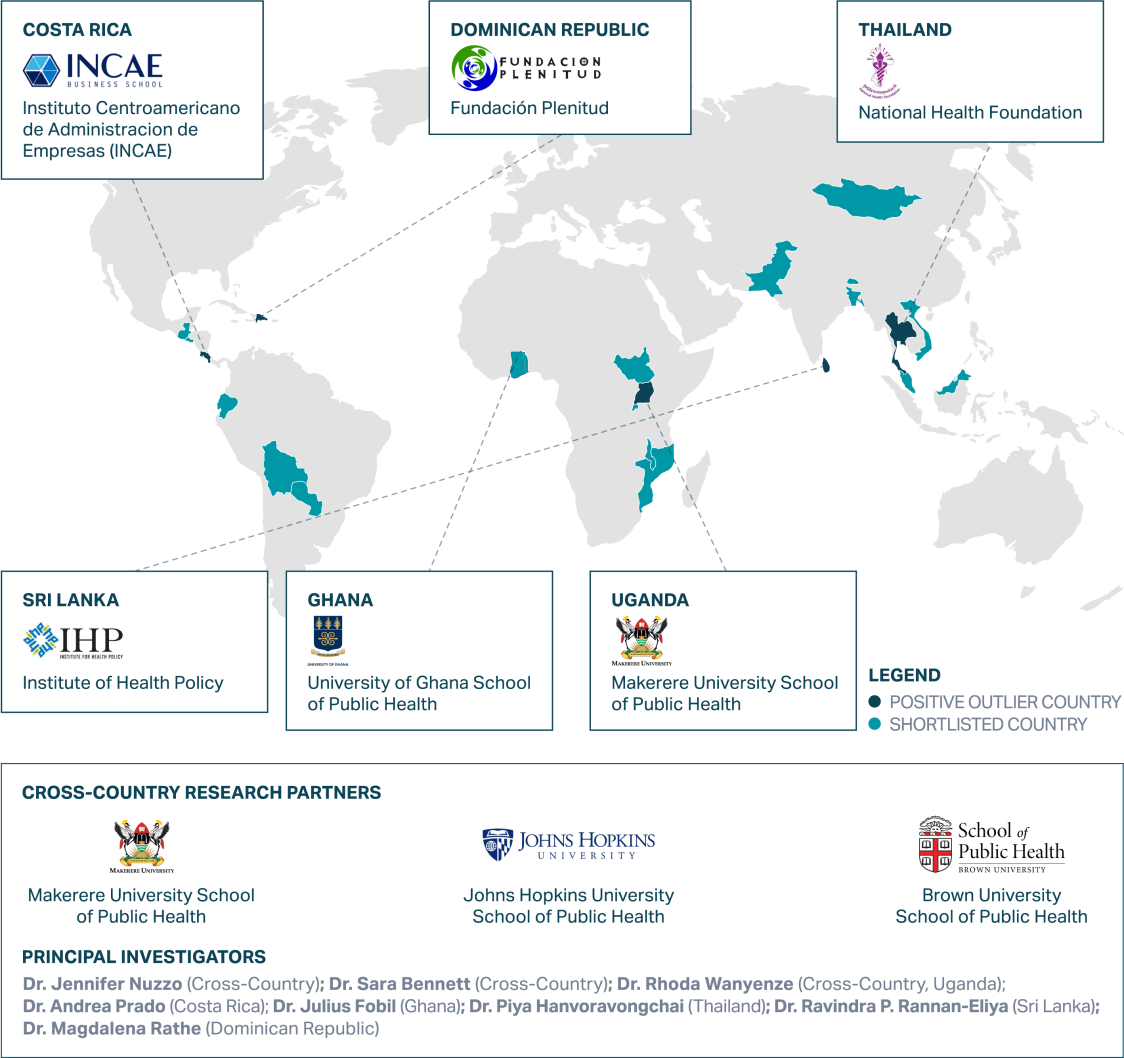
Exemplars in COVID-19 Response research consortium. Creator: Exemplars in Global Health.

We conducted the study in two phases. Phase 1 identified countries that demonstrated evidence of successful pandemic response and the ability to maintain EHS. Six countries from Asia, Latin America and Sub-Saharan Africa (two from each region) were identified as having more robust performance compared with their peers based on a selection of quantitative COVID-19 response indicators (including age-standardised death rates, COVID-19 cases per capita and COVID-19 test positivity rate) and EHS indicators (including disruption to diphtheria-tetanus-pertussis immunisation). Given the lack of accessible, reliable and consistent COVID-19 data, a wide range of countries that had performed relatively well on COVID-19 indicators were considered. We supplemented our quantitative analysis with policy and literature reviews and key informant interviews (KIIs) of regional stakeholders to examine in-country policies and strategies implemented and further understand countries’ experiences during the pandemic. Additional factors were considered, such as the availability of good quality data and the transferability of the findings. Priority was placed on countries that represented different governmental structures or systems and different geographical locations within the region (ie, non-neighbouring countries). The existence of an ICRP who can lead deep-dive research efforts and have the local expertise to identify beneficial practices in their countries was also considered. The ICRPs for the ECR research study are reflected in figure 1.

Phase 2 involved deep-dive research efforts in each country to identify best practices, policies and lessons learnt for COVID-19 response and maintenance of EHS. This included desk reviews, KIIs of key stakeholders and quantitative and qualitative data analysis (for more details on the methodology of each phase, please refer to the Mullen *et al* methods paper and country-specific papers included in the BMJ Global Health EGH supplement. Project details are also published on the EGH website: https://www.exemplars.health/emerging-topics/ecr/cross-country-synthesis/methodology).

In regard to the definition of EHS, while the researchers acknowledge the broad definitions provided by the WHO,^16^ ICRPs were encouraged to use context-specific definitions of what services are considered ‘essential’ by health authorities in their countries, especially during a crisis. Based on WHO’s scoping review of EHS provision during disruptive events, our research primarily focused on country policies and practices that addressed (a) decreased provision of health services, (b) increased and emerging healthcare needs and (c) the need to adapt health service delivery, such as minimising face-to-face contact.^16^

The study was guided by a Technical Advisory Group (TAG), consisting of a diverse range of technical experts and advisors from the Africa CDC, Bill & Melinda Gates Foundation, Harvard School of Public Health, National Centre for Infectious Diseases in Singapore, Resolve to Save Lives, Pan American Health Organization (PAHO), World Bank and the WHO.

**Figure 2:**
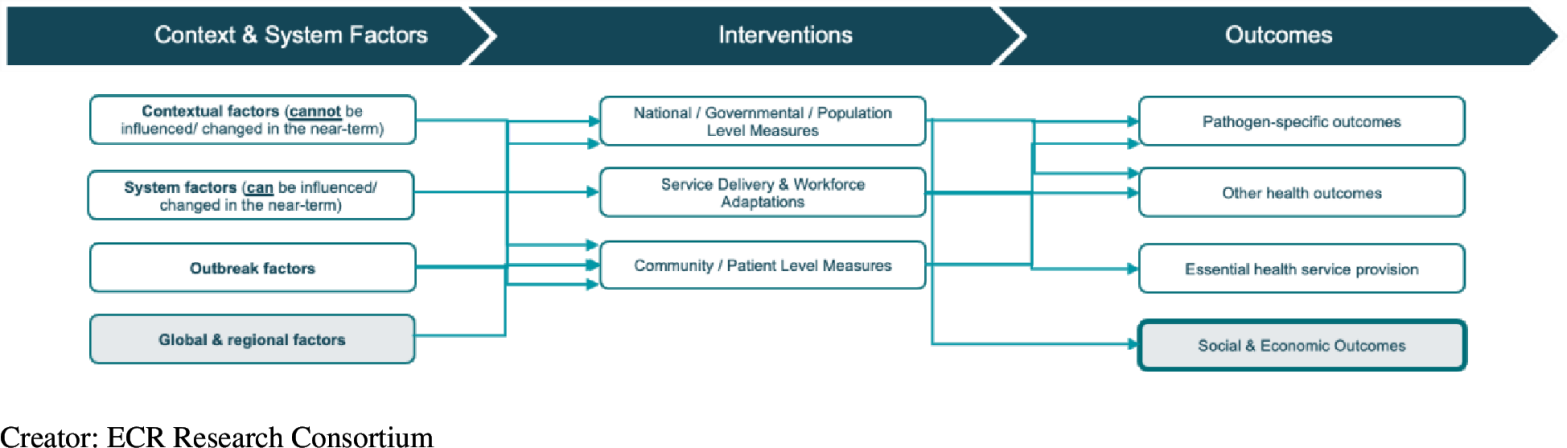
Exemplars in COVID-19 Response conceptual framework.

The research partners developed a conceptual framework to scope and guide research and to identify and classify beneficial policies and practices in each of the six countries. The ECR conceptual framework (figure 2) categorises countries’ responses in three areas:

- Contextual and system factors: Each country started the pandemic with contextual and system factors that could impact the trajectory of COVID-19 within their borders. Examples include health system factors such as healthcare accessibility, affordability and underlying disease burden, in addition to unique political, economic, social and cultural factors that may have influenced the country’s pandemic response or continuation of EHS.
- Interventions: The interventions that each country put in place to combat COVID-19 and maintain EHS can be further broken down into population-level, health system-level and patient-level interventions. Many of our KIIs focused on how countries chose to implement and prioritise certain interventions over others. Not all subdomains in this area were explored in depth in this study as they may not have been prioritised in our selected countries.
- Outcomes: Outcomes and impacts of each country’s interventions and contextual factors during COVID-19 were examined using desk review, KIIs and both quantitative and qualitative sources.

This conceptual framework was used to guide in-country research and as a foundation for this cross-country synthesis. ICRPs used the framework to scope and classify their desk research and KIIs. The CCRPs also used a modified version of the conceptual framework to later categorise policies and practices that were employed by each of the six countries and to classify them as beneficial.

ICRPs performed mixed-methods research to identify beneficial practices employed by their country for COVID-19 response and EHS delivery. Throughout the project, ICRPs regularly shared their deep-dive research findings with the research consortium and TAG to identify emerging cross-country themes and policy relevant findings. Each ICRP developed a final report of the national efforts to maintain EHS during the pandemic, including beneficial practices, enablers and barriers, as well as lessons learnt and recommendations for strengthening health system resilience and pandemic preparedness and response (PPR).^17^,^21^

### Cross-country analysis to identify beneficial practices and policies

To identify beneficial practices across all six countries, Brown SPH and JHSPH led a cross-country analysis of the deep-dive research performed by ICRPs. At least two researchers extracted and thematically coded beneficial practices/policies, enablers and barriers for COVID-19 response and/or maintenance of EHS from the ICRP final reports. The cross-country themes that were identified were organised into three categories based on governmental/health system levels in the ECR conceptual framework:

- National/governmental/population-level measures
- Measures for service delivery and workforce adaptations
- Community and patient-level measures

The full research team (CCRPs in coordination with each ICRP) reviewed the coded information for accuracy and country representation. For each beneficial practice/policy theme, we tallied the number of countries that showed evidence of having employed it based on ICRP research and final reports.

We considered practices or policies to be beneficial if there was thematic evidence that the policy or practice was employed and reported as beneficial by three or more countries. Common cross-cutting enablers and barriers to response efforts were identified. The cross-country analysis focused on commonalities between countries, rather than differences, for three reasons. First, variations in each country’s final report and methodology meant that it could not be assumed that the absence of a theme was due to a real absence of such activities in the country. Second, evidence that a practice or policy was employed by multiple countries increases the likelihood that it would be replicable in other settings. Third, evidence that multiple countries reported a practice or policy as beneficial increased our confidence in the importance, and possible exemplary nature, of that measure.

Thematic commonalities of beneficial practices were validated through a group review between the lead researchers, ICRPs and Gates Ventures and presentation to the TAG. Brown SPH and JHSPH researchers synthesised cross-cutting themes, country experiences and lessons learnt to develop key insights and best practices on health system resilience and preparedness for future health emergencies that may be applicable to comparable settings.

#### Patient and public involvement

There was no direct patient involvement in this project. The public has been involved through KIIs and dissemination at international, regional and local conferences and meetings. Webinars have also been hosted to share country-specific findings with in-country stakeholders. KIIs have included government officials, public health authorities, healthcare providers, civil society and non-governmental organisation (NGO) staff, and other stakeholders. There is also an EGH public website hosted by Gates Ventures that presents country narratives and interactive graphics for lay audiences.

As shown in table 1, a total of 13 cross-cutting themes and beneficial practices emerged from the cross-country analysis. All themes applied to three or more (out of six) countries and are organised here by each of the governmental/health system categories described above.

**Table 1:** Cross-cutting themes and beneficial practices identified from the cross-country synthesis

Health system level	Cross-cutting theme/beneficial practice	ECR countries					
		Costa Rica	Thailand	Dominican Republic	Sri Lanka	Uganda	Ghana
National/governmental/population-level measures	A whole-of-government response with multisectoral collaboration and early establishment of national decision-making bodies (N=5)	☑	☑	☑	☑	☑	
	Early, effective outbreak control and case management to suppress COVID-19 transmission and minimise disruption to EHS (N=6)	☑	☑	☑	☑	☑	☑
	Partnerships with the private sector (N=6)	☑	☑	☑	☑	☑	☑
	Academic-government partnerships facilitated data-driven decision-making (N=6)	☑	☑	☑	☑	☑	☑
	Existing financing mechanism(s) dedicated to supporting emergency response and EHS (N=6)	☑	☑	☑	☑	☑	☑
	Expansion of health financing schemes to increase care coverage (N=5)	☑	☑	☑	☑		☑
	Government policies/initiatives in place to prioritise the maintenance of EHS (N=5)		☑	☑	☑	☑	☑
Measures for service delivery and workforce adaptations	Service delivery adaptations and provision of care through alternative modalities to minimise disruption of EHS (N=6)	☑	☑	☑	☑	☑	☑
	Rapid mobilisation and deployment of health workforce for COVID-19 response and EHS delivery (N=6)	☑	☑	☑	☑	☑	☑
	Operational flexibility to transfer patients and resources between health facilities (N=4)	☑	☑	☑	☑		
	Adaptation of existing health infrastructure and disease response capacities (N=6)	☑	☑	☑	☑	☑	☑
Community and patient-level measures	Mental health and psychosocial support programmes for the community (N=5)	☑		☑	☑	☑	☑
	Robust community engagement efforts (N=5)	☑	☑		☑	☑	☑

Creator: M. Guha and A. Cohen Tanugi-Carresse.

EHS, essential health services.

## National/governmental/population-level measures

### A whole-of-government response with multisectoral collaboration and early establishment of national decision-making bodies

A key cross-cutting theme identified across five countries was the coordinated whole-of-government response with multisectoral collaboration involving non-health sectors and agencies, which contributed to responding effectively to the pandemic and maintaining EHS. Countries also underscored the importance of early establishment of national decision-making bodies championed by senior-level government leaders to facilitate swift action and policy decisions during a rapidly evolving health emergency.

Thailand’s initial national COVID-19 response was led by a centralised, multisectoral leadership body and command centre in the Centre for COVID-19 Situation Administration (CCSA), which was established and chaired by the Prime Minister in March 2020. The members of the CCSA were top-level administrators from all ministries who contributed to a ‘whole-of-government’ and ‘whole-of-society’ response, taking into account the health, economic, political and social impacts of the pandemic. The CCSA was essential for high-level decision-making and coordinated response efforts. Proposals were submitted to the CCSA for decision, endorsement and implementation nationwide (primarily through coordination of provincial governors), ensuring unified command and integrated response and collaboration across all agencies. This centralised structure was supported by legal frameworks that facilitated the swift enactment of COVID-19 response functions and mandates, as well as the rapid mobilisation of budgets and human resources required to meet the increasingly complex demands of the pandemic. Respondents attributed Thailand’s successful COVID-19 response and ability to maintain EHS to the country’s strong leadership and governance structures and long-standing investments in health system strengthening and preparedness – key enablers identified in this project.

Several study countries stressed the importance of early establishment of national, multisectoral decision-making bodies and early activation of the Emergency Operations Center (EOC) and Incident Command System (ICS). Uganda’s pandemic response was championed at the highest levels of government, with the President chairing the multisectoral National Task Force (NTF), composed of political and technical leaders from key government sectors and agencies. Both Uganda and Thailand activated EOCs/ICS at multiple levels as early as January 2020.

In Costa Rica (CR), the National Situation Analysis Room of the EOC consisted of multi-institutional partners including the Ministry of Health (MoH), the CR Social Security Fund (CCSS), the National Emergency Commission, the Ministry of National Planning and Economic Policy, the Ministry of Economy, PAHO, the Joint Institute for Social Assistance and the INCAE Business School. In Sri Lanka, the National Operations Centre for Prevention of COVID-19 Outbreak, a multi-agency team responsible for managing the pandemic and resultant social issues, was established in March 2020 and led by the Army Commander, reporting directly to the president. The country’s multisectoral approach to COVID-19 consisted of diverse stakeholders from health, military, law enforcement and the private sector. The early and planned prioritisation of multisectoral collaboration between health and non-health agencies and establishment of national decision-making bodies were essential to support coordinated response efforts and maintenance of EHS in study countries.

### Academic-government partnerships facilitated data-driven decision-making

Multiple countries established partnerships between academia and government which facilitated data-driven decision making and policy translation during the pandemic. Thailand’s robust academic and research networks were highly active during the pandemic, documenting and facilitating data-driven decision-making at all levels. Strong connections between local research entities and health decision-making bodies allowed for collaborative approaches to regularly analyse COVID-19 surveillance data, inform policymakers and provide guidance on prevention and control measures.

In March 2020, the Ugandan NTF established a scientific advisory committee made up of interdisciplinary public health specialists and academicians from MakSPH and schools of medicine and statistics, the Medical Research Council and the Uganda Virus Research Institute to collate, synthesise and interpret emerging data and translate new information into evidence-informed policies and strategies for pandemic response. Similarly, researchers from universities in CR were actively involved in COVID-related data analyses that were presented weekly to the President of the Republic. These analyses were reviewed as part of the multi-institutional decision-making process for pandemic response within the EOC.^22^ In the Dominican Republic (DR), the Technological Institute of Santo Domingo developed a predictive model for the epidemiological behaviour of COVID-19 to manage data on cases, mortality, non-pharmaceutical interventions (NPIs) and health planning in order to monitor the pandemic. This model was used by the government to help with case management and reduce the burden of hospitalisations.

### Partnerships with the private sector

All six countries highlighted the importance of partnerships with the private sector in responding effectively to the pandemic and maintaining EHS. Various government collaborations with the private sector facilitated the procurement of personal protective equipment (PPE) and other resources, scale up of testing and vaccinations, case management, maintaining supply of medicines to patients in the community, and/or the provision of EHS.

One of CR’s largest public-private partnerships (ALEPP), involving multisectoral collaboration between the CR Chamber of Commerce, Academia and the CR Social Security Fund, significantly contributed to the procurement of necessary resources during the pandemic. ALEPP coordinated the CR value chain to produce PPE in the country; they identified local companies, various production capacities and necessary inventory and raw materials. As a result of this initiative, 600 000 face shields were manufactured locally by Grupo Vargas, which transformed its operations into plastic to adapt to the increasing PPE demand.^22^ The collaboration also identified protocols to decontaminate N95 masks using resources that local companies had available in their production plants.

The DR leveraged past PPR plans to develop their COVID-19 contingency plan, which laid the groundwork for public-private partnerships (PPPs) in the country. Multisectoral coordination between the government and the private sector facilitated the procurement of hospital resources and strengthened testing capacity and the roll-out of an effective vaccination campaign. Leveraging DR’s existing private laboratory system, the government was able to make all testing and treatment free-of-charge in all sectors during the first year of the pandemic. Private companies also helped finance vaccine purchases, manage vaccine storage and transport and provide physical space for vaccination centres. Private sector investment and support was credited with helping the DR government procure vaccines early and vaccinate 77% of the adult population by May 2022.

Reliance on PPPs also contributed to Uganda and Ghana’s successful pandemic response. In order to support the overwhelmed government testing centres, private laboratories in Uganda were licensed and permitted to provide additional testing services. Partnerships with private companies, NGOs and foreign governments enabled the procurement and manufacturing of essential goods and PPE for healthcare facilities and providers. In Ghana, a coalition of civil society, community-based organisations, private sector entities such as banks and pharmaceutical companies, and philanthropists was activated to form an alliance that supported the procurement of PPE, test kits and other resources needed to contain the pandemic. This made it possible to mobilise resources not just for health personnel but also for vulnerable and immunocompromised individuals during the early stages of the pandemic.

### Early and effective outbreak control and case management to minimise disruption to essential health services (EHS)

All study countries underscored that early and effective outbreak control and case management helped suppress COVID-19 transmission and minimise disruption to EHS. Given their experience with managing prior epidemics, local governments and public health authorities recognised the need to act swiftly to contain the spread of COVID-19. Countries implemented various outbreak control measures including robust testing, contact tracing and isolation efforts, targeted border control measures, public mobility restrictions, risk communications, masking and vaccinations once they became available. As a result, health systems were able to manage their COVID-19 case load while maintaining EHS.

Enforced through the Communicable Diseases Act of 2015 and multiple emergency decrees, the Thai Government used a number of mandates, policies and NPIs to suppress COVID-19 transmission including public mobility restrictions, border control, masking, surveillance and contact tracing, and risk communication efforts. Thailand implemented a robust surveillance system that leveraged foundational infrastructure established during prior epidemics. Networks of surveillance teams, including the surveillance and rapid response teams, allowed for rapid deployment of health workers to support detection, isolation and treatment of active cases. At the subdistrict level, the national network of Village Health Volunteers (VHVs) played a significant role in COVID-19 surveillance, given their reach and familiarity with local communities. Sri Lanka benefited from having strong local level competencies in surveillance and contact tracing, reflecting its previous successes in eliminating infectious diseases (IDs) such as malaria and measles. Over 2800 Public Health Inspectors were deployed to lead surveillance, contact tracing and isolation efforts. Moreover, military assistance was used to build quarantine facilities, run community vaccination centres, conduct mobile vaccination and disinfection drives, and support the police in implementing curfews and travel bans. Border control, mandatory masking and social distancing measures were also enforced.

In March 2020, Uganda activated district task forces to coordinate subnational and local COVID-19 response activities like surveillance, contact tracing and isolation. The Incident Management Team managed the country’s surveillance and laboratory systems, ensured logistics for supplies and transport and deployed rapid response teams in local districts. In Ghana, in order to enhance COVID-19 testing capacity and to maintain the integrity of samples from remote areas, drone technology was employed to shuttle COVID-19 test samples from rural areas to laboratories in Accra and Kumasi. Approximately 15 000 test samples were transported in a day, and results were delivered via text message/SMS.

All study countries recognised the importance of robust and effective public communication during the COVID-19 response. They emphasised the need for trusted messengers and consistent messaging through well-coordinated daily briefings, use of social media and traditional media platforms, and helplines to keep communities informed and maintain EHS. Stakeholders such as government officials, public health experts, health workers and multisectoral partners participated in regular briefings to discuss COVID-19 measures and adaptations to EHS. By establishing open communication channels with the public, these countries built trust and promoted adherence to COVID-19 measures and the utilisation of EHS.

#### Robust vaccination efforts

Several countries employed robust COVID-19 vaccination efforts to help reduce the burden on health systems and ensure health workers and resources were available to provide EHS. Government collaborations with international agencies and multisectoral partners including the private sector were essential to procure vaccines and ramp up vaccination efforts. Please refer to online supplemental text for country examples.

### Existing financing mechanism(s) dedicated to supporting emergency response and EHS

Another cross-cutting theme identified across five study countries was the existence of financing mechanisms dedicated to supporting emergency response and the maintenance of EHS. This provided countries with the flexibility to adapt to the rapidly evolving and complex needs of the pandemic.

In 2016, CR’s CCSS Board of Directors established a contingency fund to provide monetary resources in case of climate disasters or epidemics. The fund also received additional investments from the Central Government and loans from international finance institutions. In 2020, the CCSS used these funds to increase hospital capacity and infrastructure, cover healthcare personnel remunerations and procure necessary resources to maintain EHS. This included adaptations like CR’s Vacancy Substitution Scheme which supported the mobilisation of healthcare workers (HCWs) from certain specialties to high-need COVID-19 facilities. The second largest expenditure was on durable goods such as purchasing new medical equipment (ventilators, intensive care unit (ICU) beds) for the COVID-19 specialised centre and other hospitals and financing remodelling and adapting the infrastructure of several hospitals across the country.

Both Sri Lanka and Thailand made emergency allocations through central budgetary mechanisms, as well as supplementing this by provision of targeted loans. In both countries, maintenance of EHS was facilitated by continuing to finance routine health services through existing budgetary arrangements, with much of the new emergency funding channelled through separate budget lines to pay for COVID-19 specific costs, including laboratory testing, vaccines and disease control activities. This dual approach to financing helped maintain the resilience of EHS. Thailand also used loans to support the public health workforce and procurement of medical supplies and vaccines. Both countries used loans to provide social support to affected and vulnerable households and businesses, but in Sri Lanka’s case, the extent of this was limited by lack of fiscal space.

Furthermore, the Thai government facilitated rapid manoeuvring of health financing mechanisms to ensure that COVID-19 operations remained funded separately from other non-COVID-19 services. This action allowed for minimal to no funding disruptions to EHS during the pandemic. These efforts were coupled with the activation of funds like the Community Health Fund to further support community-based HCW networks to support both COVID-19 and non-COVID-19 services. Without this financing, EHS providers may not have been able to devote time and resources to adapting and innovating service delivery in the face of numerous challenges presented by the pandemic.

Please refer to online supplemental text for details on the expansion of Universal Health Coverage and/or other health financing schemes to increase care coverage during COVID-19.

### Government policies and initiatives in place to prioritise the maintenance of essential health services

During the early phase of the pandemic, country governments put in place policies and initiatives to prioritise the provision of EHS such as maternal and child health and NCD services, recognising the need to ensure optimal care is given to non-COVID-19 patients. In April 2020, the Ugandan MoH established a committee specifically focused on the continuity of EHS. Members included MoH officials, district government representatives, public health authorities and international partners such as the WHO and UNICEF. The committee oversaw all efforts to maintain access to—and adapt service delivery for—routine and EHS.

In Thailand, the Department of Medical Services under the MOPH established a national ‘New Normal Medical Services’ initiative that was implemented across all levels of the health system, with the goal of ensuring both COVID-19 and non-COVID-19 patients receive appropriate treatment and care. This consisted of a package of innovative approaches including digital health solutions to reduce crowding and control infections in healthcare settings while also promoting equity. Under the New Normal Medical Services and informed by pilot innovations, a comprehensive set of practices was designed and implemented across the country specifically focused on maintaining NCD services. The NCD redesign model consisted of population management and clinical risk stratification to focus efforts on high-need patients; greater empowerment of patients through digital health and self-monitoring; and home/community delivery of medications (eg, by mail). Additionally, the Thai government employed a number of strategies to maintain routine immunisation services during the pandemic including: (i) separate well-baby clinics in hospitals to separate the sick from the healthy, (ii) non-traditional vaccine venues (eg, drive-thru options or vaccination at home), (iii) public communication efforts and (iv) catch-up campaigns.

DR leveraged past PPR plans to create and enforce their contingency plan, which emphasised both the importance of effective outbreak control and maintenance of EHS. With technical support from PAHO, the US Centers for Disease Control and Prevention (CDC) and the United States Agency for International Development, the DR government was able to implement the contingency plan quickly and produce the ‘General Guidelines for the normalisation of the provision of health services in the face of the COVID-19 pandemic’ in June 2020 which underscored the importance of maintaining EHS in the midst of a health emergency. Similarly, Sri Lanka’s COVID-19 Preparedness and Response Plan published in April 2020 specifically recognised the maintenance of EHS as a priority during the pandemic (refer to online supplemental text for more details).

## Measures for service delivery and workforce adaptations

### Service delivery adaptations and provision of care through alternative modalities

Several service delivery adaptations were implemented across study countries to support COVID-19 patients while maintaining access to EHS. In order to minimise disruptions in care delivery and adhere to infection control protocols, efforts were made to separate COVID-19 care from non-COVID-19 care, limit the number of non-urgent in-person interactions and reduce chances of patients contracting COVID-19 in the healthcare setting. Some countries built new field hospitals as emergency projects (Ghana), while other countries like Thailand and Sri Lanka repurposed non-health venues (eg, hotels, conference centres) into COVID-19 care facilities. In collaboration with the private sector, the Sri Lankan government established intermediate care centres in hotels across the country serving patients with mild to moderate COVID-19 symptoms in order to reduce the burden on public facilities. The military also built quarantine centres in key areas with MoH support. In March 2020, anticipating the high demand for hospitalisation services and ICU beds, CR repurposed rehab centres into hospitals dedicated to caring for COVID-19 patients.

Thailand employed a number of service delivery adaptations to maintain access to EHS. A primary way NCD services were maintained was through the ‘New Normal Medical Services’ initiative as described above. The model leveraged existing infrastructure to make efficient use of limited resources, focusing on patient-centred services, with care devolved at the community level. This was done through mobilising existing VHV networks, building off years of investment into primary care systems and health personnel recruitment.

Please refer to online supplemental text for country examples on digital health solutions that were implemented during COVID-19.

### Rapid mobilisation and deployment of health workforce for COVID-19 response and EHS delivery

During the pandemic, several countries hired additional HCWs and/or redeployed existing HCWs to high-burden hospitals and communities. Task shifting and re-assigning of roles/responsibilities were also employed to cover COVID-related functions and ensure staff were not overworked, burned out and/or overexposed to COVID-19.

CR, Thailand and Uganda hired both temporary and full-time personnel of all types—including doctors, epidemiologists, anaesthetists, nurses, laboratory technologists and ambulance drivers—to maintain EHS and provide COVID-19 care. Some were deployed to dedicated COVID-19 treatment centres and/or Points of Entry for surveillance. In Uganda, community health workers (CHWs) and medical and public health students contributed to efforts of contact tracing and provision of EHS. Uganda and Thailand mobilised and trained their village health worker networks to serve local communities. Sri Lanka mobilised field staff from other health programmes (eg, mosquito-borne disease control) and medical officers from non-clinical areas to help manage the outbreak, allowing EHS staff to continue providing services.

Thailand, CR and Uganda reported limited access to some types of specialist providers, such as critical care physicians and ICU nurses, highlighting the need for countries to invest in training specialists. In early 2020, 10 out of 77 provinces in Thailand accounted for 87% of total COVID-19 cases. This prompted the MOPH to mobilise surplus capacity from low burden provinces, as a shortage of specialists, in particular ICU nurses, critical care experts and epidemiologists, became evident in certain locations. In provinces with a high caseload and critical shortage of HCWs, medical teams were mobilised from other provinces and provided on-the-job training as needed for critical care and infection prevention and control (IPC). Sri Lanka used an existing national monitoring mechanism of ICU beds to transfer patients within the island when needed.

The national governments of Thailand, DR, Uganda and Ghana provided financial support/monetary incentives and social support to HCWs supporting pandemic response to improve job satisfaction, enhance performance and recognise their hard work. The DR government provided a 30% increase in salaries for all doctors, nurses and bioanalysts. 36% of CR’s contingency fund was used to finance remunerations, including new job positions, paying for extra hours and the accompanying social security costs. The Thai government converted approximately 40 000 contract-based health workers to permanent civil service positions and provided lump sum payments to healthcare workers or their families who contracted COVID-19. They also introduced compensation for ‘hazardous’ work and developed national social recognition campaigns for health workers.

### Operational flexibility to transfer patients and resources between healthcare facilities

Another beneficial practice was the transfer of patients, resources and supplies between healthcare facilities in real-time depending on patient needs and capacity. In April 2020, the DR government integrated the Command, Control, Communications, Computers, Cybersecurity and Intelligence Center of the Ministry of Defense with the health system, specifically the National Health Service and the MoH. They developed a digital platform that centralised data from hospitals, clinics, laboratories, pharmacies and insurance providers and presented in real time the number of beds (including ICUs), ventilators and ambulances available and in use in different locations as well as other data that contributed to predictive models to support decision-making. The platform was most useful in ensuring that large hospitals did not reach max capacity. There was also an artificial intelligence system that made projections and developed epidemiological profiles in order to predict the behaviour of the virus and inform policies.

Both CR and Sri Lanka benefited from having a highly integrated healthcare delivery system with relatively strong central management and coordination. In CR, a single institution, the CCSS, controls the entire national network of public clinics, hospitals and centres, while in Sri Lanka, its central health ministry has effective de facto control of all public health facilities, including those run by provincial councils. This allowed both countries to establish coordinated pandemic response plans and efficiently respond to regional surges. During the pandemic, national coordination taskforces in Sri Lanka and CR met weekly to make real-time health system capacity decisions—they managed deployments on a daily basis, leveraging digital information systems to monitor factors such as bed occupancy rate by health facility and patient transfers, ensuring that hospitals did not reach max capacity. The digital health platform ‘Expediente Digital Único en Salud’ was an essential tool that provided government decision-makers with real-time data in CR.

### Adaptation of existing health infrastructure and disease response capacities

Given prior experience responding to epidemics such as SARS, H1N1, MERS, HIV and Ebola, many of the study countries were able to quickly leverage and adapt existing disease response capacities and infrastructures including rapid response teams, lab transport networks and coordination structures. Public health authorities in these countries recognised the need for comprehensive preparedness, developed capacity to respond to IDs, and rapid decision-making and action to manage outbreaks. Both Sri Lanka and Thailand are good examples of this. Both countries had existing networks of public health surveillance teams, staffed by public health nurses and officers, based at the local level, with substantial experience in disease surveillance and control, including contact tracing. In Sri Lanka, the teams were based in 400 local health units, and in Thailand, over 1000 surveillance and rapid response teams were positioned across the country to rapidly respond to health threats. During the pandemic, both countries deployed these teams to isolate cases, provide treatment, and actively trace and quarantine contacts. Additionally, Thailand’s long-established national Field Epidemiology Training Program had trained thousands of experts in disease outbreak investigation and control, many of whom were stationed at the provincial and district level to manage COVID-19 outbreaks and conduct contact tracing and epidemiological analyses.

## Community/patient level measures

### Robust community engagement efforts

Multiple countries leveraged existing CHW networks for community engagement on COVID-19 response and access to EHS. CHWs played an important role in COVID-19 surveillance, testing and contact tracing, risk communication, vaccination and provision of primary care services to communities that were in ‘care deserts’. Thailand’s national network of VHVs played a significant role in COVID-19 surveillance and community engagement, given their reach and familiarity with local communities. In addition to contact tracing, data analysis and risk communication, VHVs enabled people to receive different types of health services close to their homes, which may have contributed to higher service utilisation and lower unmet needs for outpatient and inpatient services. According to one study, VHVs went to more than 14 million households between March and April 2020. VHVs also identified and monitored over 809 000 unemployed workers returning to their hometowns.^20^

Similarly, Uganda and Ghana leveraged existing disease response capacities to fight COVID-19. Uganda deployed more than 10 000 CHWs (locally referred to as village health teams) established during previous epidemics and trained on IPC, epidemic surveillance and other aspects of outbreak response. In order to reach rural/underserved communities, CR involved the directors of ‘health areas’ and their army of CHWs to carry out vaccination campaigns in remote areas.

### Mental health and psychosocial support programs for the community

With prolonged lockdowns, quarantines, curfews and social distancing, the mental health and well-being of communities and individuals became a growing concern in many countries. Several countries implemented programmes and policies to increase awareness, decrease stigma and support individuals struggling with mental health issues. In CR, the MoH, College of Psychology Professionals and the 911 Emergency System joined forces to establish the ‘Psychological Support Office,’ which provided a 24-hour hotline for people needing mental health support. Sri Lanka prioritised promoting mental health and psychosocial well-being early in the pandemic by extending the National Mental Health Helpline to all districts. The Directorate of Mental Health of the MoH, the Sri Lanka College of Psychiatrists, the WHO, and the Mental Health and Psychosocial Support provided continued access to essential mental health services and medications throughout the pandemic. Guidelines for health administrators to promote the mental well-being of frontline health workers and curtail future mental health conditions were also developed by the DMH. Furthermore, DR focused its efforts on providing follow-up care for COVID-positive individuals via the COVIDRD contact-tracing application, which also included mental health advice.

Some study countries targeted specific communities—for instance, Ghana provided support programmes for healthcare workers’ well-being while Uganda focused on children’s mental health. The NGO Save the Children provided psychosocial support through regular phone calls, home visits, mini gamebooks and radio messages to reduce stress among children. Additionally, the Obuntu Bulamu, a public project which aims to improve participation, inclusion and quality of life for children with disabilities in Central Uganda, implemented a peer-to-peer support intervention for those children, along with their non-disabled peers, parents and teachers. Across the country, CHWs were trained and encouraged to provide psychosocial support to patients.

Additional country examples for each cross-cutting theme are provided in online supplemental table 2.

## Cross-cutting enablers and barriers

This research highlighted various cross-cutting enablers and barriers that facilitated or hindered country efforts to successfully respond to COVID-19 while maintaining EHS. The following were identified as common enablers across multiple study countries:

- Long-standing investments in health systems strengthening (HSS) and preparedness leading to stronger disease response capacities, a robust and skilled workforce, engagement with communities and sustained financing mechanisms.
- Pre-crisis integration of the health system allowing for operational flexibility and integrated care delivery.
- High degree of public trust in government and health system leading to social cohesion and adherence to public health measures.
- Strong leadership, political will and governance structures that allow for multisectoral collaboration and decision-making and rapid implementation of national policies.
- Prior experience with responding to epidemics led to knowledge continuity between healthcare providers, government officials and other responders.
- Strong primary healthcare systems capable of managing pandemic response and EHS delivery.
- Substantial progress towards universal health coverage (UHC) or other health financing schemes.
- Existing partnerships with the private sector and/or international agencies that could support the provision of healthcare services or response operations such as PPE/vaccine procurement and financing.
- Use of electronic health records and other digital tools to support surveillance and enable an integrated response.
- Provision of social and economic support to frontline workers, the unemployed and underserved communities.

Numerous countries cited the benefits of their prior experience responding to epidemics including SARS, MERS, Ebola and malaria, applying these lessons learnt for early outbreak control. Thailand, Uganda, Ghana and Sri Lanka noted the health investments and infrastructure put in place prior to COVID-19 for disease surveillance, contact tracing, skilled workforce and community engagement efforts. Sri Lanka’s national immunisation programme was also very strong, with coverage levels for children higher than most high-income countries which facilitated COVID-19 vaccination efforts and wide public acceptance and uptake.

Thailand and CR’s long-standing investments in HSS have led to integrated and responsive health systems with a large skilled workforce, strong primary healthcare systems and sustained health financing mechanisms. Given most of CR’s public health system is overseen by a single entity (CCSS), this integrated network management system and centralised approach to healthcare delivery enabled the country to maintain access to both emergency and EHS throughout the pandemic. Ghana similarly reported the benefits of sustained investments in health which contributed to their success in maintaining EHS and responding to COVID-19, particularly through the adoption of UHC and being a leading producer of doctors, midwives and nurses.

Study countries also identified cross-cutting barriers and challenges that hindered their ability to maintain EHS and/or effectively respond to the pandemic. First, there was no clear context-specific definition for EHS which led to confusion around which services were considered ‘essential’ and should therefore continue operating during the pandemic. Second, multiple countries reported the lack of real-time, high-quality, stratified and accessible health data which impeded evidence-informed decision-making on response efforts as well as monitoring of EHS and health outcomes. Third, the digital divide and inequitable access to technology and equipment among underserved communities limited the effectiveness of telehealth services. Many physicians reported doubts about the quality of care provided through telemedicine due to the lack of experience using such tools, the lack of quality control and limited access to technology among low-income patients.

## Discussion

As the COVID-19 pandemic has demonstrated, acute-onset ID emergencies can affect population health in multiple ways by stressing existing health system capacity and interrupting the provision of essential services for other population health needs. Efforts to contain the spread of the ID may directly, through EHS visit cancellation, or indirectly, through patient avoidance, reduce the provision of health services and further worsen health outcomes, resulting in excess morbidity and mortality from preventable causes.^1 2^

Recognising the need for countries to be ready to respond to multiple competing health demands during a public health emergency, this analysis sought to identify generalisable policies and practices that may aid countries’ efforts to strengthen health system resilience and maintain EHS while also responding to an ID emergency. Specifically, this analysis examined six LMICs’ responses to the COVID-19 pandemic to identify what policies, practices and resources were used and thought to be beneficial in enabling countries to continue to provide EHS to their populations, while also meeting the acute care demands of the health emergency. In this study, we identified common themes across multiple countries in order to determine beneficial practices that may be applicable in other comparable settings. Ideally, other countries will consider these practices as they develop and refine their plans to strengthen health system resilience and preparedness for future public health emergencies.

Although this analysis did not attempt to evaluate the effectiveness of countries’ COVID-19 response or the specific strategies that countries employed to slow or stop the spread of COVID-19, a common theme that emerged across all six countries is that limiting the spread of COVID-19 early on helped create more bandwidth within the health system to meet the acute demands of the pandemic, as well as to continue to provide other EHS. How countries responded to COVID-19 may have had differential impacts on the need for and availability of EHS. Other themes that several countries deemed critical to their response efforts included whole-of-government approaches, multisectoral collaboration and decision-making, partnerships with the private sector, health financing mechanisms, a robust health workforce, community engagement and service delivery innovations such as digital health.

Our findings are consistent with recent literature on learnings from COVID-19 and building health system resilience.^24^,^6 23^ ^26^ In a 2023 paper, Mghamba *et al* present case studies from five Commonwealth countries (Guyana, Malawi, Rwanda, Sri Lanka and Tanzania) that focus on how these countries used innovative, integrated approaches to build health system resilience and maintain EHS during COVID-19. Key strategies included digital tools, improvements in emergency risk management, developing centralised governance and coordination mechanisms and multisectoral partnerships, and strengthening surveillance and community engagement—themes that are also evident in our research.^24^ Similar country examples have been documented from diverse geographical and developmental contexts, further illustrating the global applicability of the resilience practices identified in our research.^2326^,^28^ While there is no one size fits all approach, the lessons derived in this paper can aid in understanding how health system resilience and preparedness can be operationalised in different contexts.

Partnering with the private sector emerged as a cross-cutting theme for rapid response to the pandemic and a vital component to strengthen health system resilience. Our findings are supported by several reports that have been published by the WHO, World Bank, Asian Development Bank, United Nations and others highlighting the importance of PPPs during the COVID-19 response.^16 29^ Country examples have been documented from Kenya, India, Singapore, South Korea and others.^29^,^31^ By leveraging the strengths and resources of both private and public sectors, these partnerships facilitated a more comprehensive and rapid response to the health crisis. The private sector’s capabilities in innovation, technology and resource mobilisation complemented the public sector’s regulatory and policy-making frameworks, leading to more efficient healthcare delivery, improved diagnostic and treatment capacities and accelerated vaccine development and distribution. While PPPs have been increasingly used as a mechanism to address global health issues, there is no agreed unifying definition of PPP; further research is needed on what makes these partnerships successful.^29^

Countries that had strong, well-integrated health systems at the outset of the pandemic had a number of advantages. These ranged from the ability to monitor healthcare demands to being able to redeploy staff, patients and resources to other facilities as demand shifted. Many of the beneficial practices reported here involved operational flexibilities and resources established prior to the pandemic. Patient trust in the health system, likely developed through the provision of quality services, also provided advantages in the overall public health response to the pandemic, such as deploying vaccines. This demonstrates that countries’ efforts to build resilient and integrated health systems will benefit them during acute public health emergencies.

A challenge in this analysis surrounds the lack of specific definitions within countries about what constitutes ‘essential health services’ and the lack of availability of real-time, high-quality data to monitor their operations. Most study countries did not have a single, clear definition of which health services are considered essential, which may have complicated efforts to monitor the pandemic’s impact on the provision of these services and implement strategies to prevent their disruption. Research teams sought data to understand the relationship between the identified beneficial practices and the availability and continued provision of EHS; however, in all cases, there was insufficient data to quantify health service impacts. Ideally, countries would identify which health services are essential and collect real-time data to monitor their provision and potential disruptions. This should be done in advance of a public health emergency to ensure the development of specific plans to minimise EHS disruption and ensure optimal health outcomes.^24^,^6^

In 2021, WHO conducted a scoping review of interventions to maintain EHS during disruptive events.^32^ ^16^ While there was no specific EHS definition given, the report includes a broad focus on maternal, newborn, child, adolescent and older people’s health (MNCAAH), including IDs, mental health and management of non-communicable diseases.^16^ In the past, WHO also included health promotion and disease prevention services and palliative care. While countries could use these reports as a reference, further research could aid in regional standardisation of EHS and guidance on data collection to monitor service delivery.

A strength of this analysis was its inclusion of six different countries across multiple geographies, each with different contexts, demographics and COVID-19 experiences. The identification of common cross-country themes, enablers and barriers helps improve our understanding of what practices may be considered by countries who wish to strengthen their health systems and improve preparedness for future health emergencies. The identification of similar approaches by separate research teams in multiple countries increases our confidence in the value of these reported practices and provides evidence that these approaches may be considered beneficial and worthy of pursuit by other countries that wish to strengthen health system resilience.

This cross-country analysis was subject to several limitations. First, the reliance on published reports and KIIs may have meant that other beneficial practices employed by countries were not documented. Similarly, because this analysis only reports the themes that were commonly identified in the country-specific reports, it may not give credit to countries for practices implemented that were not documented by ICRPs or were documented only in one or two countries. Therefore, this analysis does not represent an exhaustive list of beneficial practices that other countries should consider implementing. Second, as KIIs were conducted at different stages in each country’s pandemic response, the content of those interviews may have been influenced by recall bias and differing conditions on the ground at the time. The cross-country themes and practices presented here represent those identified by ICRPs as being beneficial in helping their countries provide EHS during the COVID-19 pandemic. Further research is needed to evaluate the effectiveness of these practices in helping prevent EHS disruptions.

Furthermore, each of the study countries had different phases of responding to COVID-19 and differing outcomes throughout the study period. These impacts may have affected the observed benefits of the practices presented in this analysis. Though the research attempted to document at what phase of response different practices were employed, this analysis was not suited to fully evaluate time-varying benefits of these practices.

Despite these limitations, this study is among the first to document operational strategies used by LMICs to maintain EHS during the COVID-19 pandemic. The reliance on ICRPs to document and analyse local, ephemeral operational information regarding countries’ responses to the pandemic and its impact on health systems is important for improving the evidence base for future preparedness efforts and to increase the diversity of perspectives included in the literature. The value of governments engaging with independent, multidisciplinary experts to vet or advise them on their response to health emergencies is a key theme that emerged during this project. In conducting this research, each of the ICRPs has established relationships with their governments and is able to advise or conduct further research on these findings. The maintenance of local research and implementation capacities to understand the impact of acute public health emergencies may serve as a future resource to governments as they develop preparedness plans and implement efforts to strengthen their health systems.

## Conclusion

The COVID-19 pandemic underscored the critical importance of robust and resilient health systems, capable of responding to acute public health emergencies while maintaining the provision of EHS. The experiences of the six countries studied highlight the value of long-standing investments in health systems strengthening and preparedness, pre-crisis integration of health systems, strong leadership and political will, existing health financing mechanisms and public trust in government. Given experience with prior epidemics, country governments acted early and aggressively to limit the spread of COVID-19 and minimise disruption of EHS. Countries were able to rapidly leverage existing disease response capacities, multisectoral coordination structures and a robust health workforce for an effective response. Whole-of-government approaches, partnerships with the private sector, service delivery innovations and community engagement were critical. Lack of context-specific definitions for EHS, inequitable access to technology and lack of access to real-time, high-quality data were common challenges and need to be addressed. These cross-country themes, enablers and barriers help improve our understanding of what practices and strategies may be considered by countries who wish to strengthen their health systems and improve preparedness for future health emergencies. Further research is needed to evaluate the effectiveness and replicability of these practices in different settings. As we move forward, it is imperative to continue research and collaboration, leveraging the lessons learnt from this pandemic to strengthen global health systems and improve our collective response to future health emergencies.

## Supplementary material

10.1136/bmjgh-2023-013392 online supplemental file 1

10.1136/bmjgh-2023-013392 online supplemental file 2

## Data Availability

Data are available upon reasonable request.
