# Supplementary material for: Maintaining essential health services during COVID-19: cross-country lessons of health system resilience from Asia, Sub-Saharan Africa and Latin America
Source: BMJ Glob Health. 2025 Oct 13;8(Suppl 6):e013392. doi: 10.1136/bmjgh-2023-013392 (PMC12826116; doi:10.1136/bmjgh-2023-013392)
Supplement: online supplemental file 1 [file bmjgh-8-Suppl_6-s001.pdf]

SUPPLEMENTARY MATERIAL

Supplementary Table 2. Country examples for all cross-cutting themes
(attached)

Supplementary Text. National / Governmental / Population Level Measures

Robust vaccination efforts

Costa Rica and DR forged partnerships with the private sector to establish vaccination centers in shopping centers, sports stadiums, passenger transportation stations, and distribute vaccines using refrigerated beer trucks. The DR MOPH leveraged existing structures responsible for administering immunizations for vaccine-preventable diseases such as the Expanded Program on Immunizations (PAI) to provide the inoculations. A digital platform ("Get Vaccinated DR") was launched to provide COVID-19 vaccine related information to the public. Moreover, private companies and small businesses also encouraged their employees to get vaccinated with the goal of re-opening the economy.

The Sri Lankan National Coordination Committee developed a detailed 'National Deployment and Vaccination Plan for COVID-19 Vaccines' in January 2021 outlining all aspects of COVID-19 vaccination from planning, regulatory, costing, prioritization, service delivery, logistics, human resources, advocacy, safety and data management. In addition to vaccination centers at public, private and military-run health facilities, mobile vaccination drives were launched to target individuals who were unable to travel to vaccination centers, including the elderly and people in nursing homes as well as large organizations like garment factories and IT companies.

Expansion of health financing schemes to increase care coverage

Another beneficial practice among four study countries was the expansion of Universal Health Coverage (UHC) and/or other health financing schemes to increase care coverage and improve access to COVID-19 care and EHS. In 2020, the DR government adopted a policy to prioritize financial protection for all, and as a result the Family Health Insurance program increased coverage from 77% of the population pre-COVID-19 to 96% by December 2020. In Costa Rica, the CCSS Board of Directors approved the extension of health insurance coverage to workers who were under labor contract suspension to support households with reduced economic income due to pandemic-related unemployment.

Many countries also ensured financial protection of underserved/minority communities who may be at higher risk of contracting COVID-19 due to the nature of their jobs, housing conditions or other social determinants of health. Thailand coupled existing UHC schemes with newly introduced health financing mechanisms in order to provide full coverage of quality COVID-19 services without co-payment. Specifically, the government expanded insurance coverage for all migrant workers. Those who work in the informal sector are covered through the country's Health Insurance Card Scheme, which is open to all documented and undocumented migrants and their dependents.

Government policies and initiatives in place to prioritize the maintenance of EHS (cont.)

Sri Lanka's COVID-19 Preparedness and Response Plan published in April 2020 specifically recognized the maintenance of EHS as a priority during the pandemic. The public sector managed by the MoH ensured EHS continued to function throughout the country mainly through the release of government circulars targeted to relevant health organizations on a regular basis. For example, in March 2020, a circular was released instructing all government healthcare institutions to continue providing treatment for patients with NCDs while adhering to COVID-19 protocols. Routine clinics providing EHS, emergency care and inpatient services for critical patients

were to remain open. Sri Lanka also implemented policies that enabled multi-month drug dispensing, utilization of postal services to deliver medicines to patients, and private sector delivery of medicines.

Recognizing the potential for disruption, the Sri Lankan MoH also took proactive measures to ensure maintenance of maternal and child health services. They specifically instructed all Medical Officers of Health to keep their clinics open, with suitable provisions made to cover for midwives on sick leave. COVID-19 safety protocols were introduced to allow home visits and clinic visits to continue. The MoH also issued guidelines that granted responsibility to the public sector to triage suspected/confirmed cases of pregnant women and newborns in designated national COVID-19 centers for management.

Supplementary Text. Measures for Service Delivery & Workforce Adaptations

Digital Health Solutions

The provision of care through alternative modalities was necessary in all countries given the health system disruptions due to COVID-19. Many hospitals were overburdened by COVID-19 patients, demand for non-COVID-19 care dropped due to fear/anxiety among patients about contracting COVID-19 at healthcare facilities, and health care providers worked from home, unable to practice at their clinics/hospitals. Patients also struggled to gain access to medications due to lockdowns. In order to limit the number of in-person interactions, all study countries employed digital health solutions to maintain EHS delivery via telemedicine, teleconsultations, and/or telemonitoring services.

Sri Lanka's leading telecommunications provider donated mobile phones and broadband routers to government hospitals and quarantine centers to coordinate patient treatment. Primary care providers in the public sector were given detailed guidelines on implementing telehealth services within their respective clinics. This included following a remote consultation algorithm to differentiate suspected COVID-19 patients from non-COVID-19 and managing them accordingly. Concurrently, medical schools and hospitals published phone numbers on their department websites, and patients were given information on how to seek medical advice through tele-consultation. Patients also used WhatsApp to send photos of symptoms, and doctors used SMS to send prescriptions to patients. Video consultation services were also made available through WhatsApp by the majority of consulting doctors at private hospitals, often through existing online appointment booking systems. Teleconsultations were mainly used for post-surgical follow-up such as cesarean sections, elective surgeries or other emergencies, and were still ongoing in 2022. Partnerships between hospitals, pharmacies and the national postal services in Sri Lanka, Thailand, Uganda and CR coordinated home delivery of medicines and multi-month drug dispensing for non-COVID-19 patients.
