# Supplementary material for: Maintaining essential health services during COVID-19: cross-country lessons of health system resilience from Asia, Sub-Saharan Africa and Latin America
Source: BMJ Glob Health. 2025 Oct 13;8(Suppl 6):e013392. doi: 10.1136/bmjgh-2023-013392 (PMC12826116; doi:10.1136/bmjgh-2023-013392)
Supplement: online supplemental file 2 [file bmjgh-8-Suppl_6-s002.pdf]

Appendix Table 2. Country examples for all cross-cutting themes

Framework Category	Cross-cutting theme/practice	Costa Rica	Thailand	Dominican Republic	Sri Lanka	Uganda	Ghana
	**A whole of government response with strong multisectoral collaboration and early establishment of national decision-making bodies**	In Costa Rica, the National Situation Analysis Room of the EOC was established and consisted of multi-institutional partners including the Ministry of Health, the Social Security Fund, the National Emergency Commission, the Ministry of National Planning and Economic Policy, the Ministry of Economy, the Pan American Health Organization, the Joint Institute for Social Assistance, and the INCAE Business School.	Thailand's national COVID-19 response was led by a centralized, multisectoral leadership body and command center in the Center for COVID-19 Situation Administration (CCSA), which was established and chaired by the Prime Minister in March 2020. The members of the CCSA were top-level administrators from all ministries who contributed to a 'whole of government' and 'whole of society' response, taking into account the health, economic, political and social impacts of the pandemic. The CCSA was essential for effective high-level decision-making and coordinated response efforts. Proposals were submitted to the CCSA for decision, endorsement and implementation nationwide (primarily through coordination of provincial governors), ensuring unified command and integrated response and collaboration across all agencies. This centralized structure was supported by legal frameworks that facilitated the swift enactment of COVID-19 response functions and mandates, as well as the rapid mobilization of budgets and human resources required to meet the increasingly complex demands of the pandemic. Thailand's successful COVID-19 response and ability to maintain EHS have been attributed to the country's strong leadership and governance structures and long-standing investments in health system strengthening and preparedness.	Creation of a National Health Cabinet, as well as other specialized bodies to address the impact of COVID-19 so that existing institutions would not be interrupted.	In Sri Lanka, the National Operations Centre for Prevention of COVID-19 Outbreak, a multidisciplinary team responsible for managing the pandemic and resultant social issues, was established in March 2020 and led by the Army Commander (Presidential Secretariat). The country's multisectoral approach to COVID-19 consisted of diverse stakeholders from health, military, law enforcement, the private sector, academics and partner organizations.<br>•In March 2020 a Presidential Task Force chaired by Mr. Basil Rajapaksa (Special Representative of the President) was formed to direct, coordinate, and monitor the delivery of continuous services as well as to sustain overall community life during the pandemic with Health, Social Services, Supply Chains, Transportation involved.<br>•In addition, several committees were set up at the beginning of the response for decision making and coordination such as the Ministry Technical Committee, Guidelines Committee, National Coordination Committee (NCC) for COVID-19 Vaccines.<br>•Although the government did not generally facilitate the involvement of non-governmental organizations in the coordination mechanism other than the business groups and health sector stakeholders, Some civil society organisations such as Sarvodaya coordinated in providing food security, hygiene and medical needs of vulnerable groups.	Similarly, the Ugandan Ministry of Health activated its Public Health EOC and National Task Force at the end of January 2020 to support and coordinate COVID-19 response efforts. The country's early response was championed at the highest levels of government, with the President chairing the multisectoral National Task Force, composed of political and technical leaders from key government sectors. In addition to the national task force, multi-sectoral district task forces were established to coordinate the response at subnational level. The district taskforces implemented the recommendations of the national task force. In June 2021, in response to Uganda's second wave of COVID-19 (largely driven by the Delta variant), the National Task Force developed a COVID-19 Resurgence Plan that divided the surveillance and laboratory pillars and proposed the establishment of new regional emergency operations centers to support district task forces.	
	**Early and effective outbreak control and case management to suppress COVID-19 transmissions and minimize disruption to EHS**	In March 2020, Costa Rica rapidly implemented nonpharmaceutical interventions and other containment measures to help delay the spread of the novel coronavirus. Weeks before the first confirmed COVID-19 death in Costa Rica, the President directed all public institutions to temporarily implement a "work-from-home" modality where possible, and on March 16th he declared the State of National Emergency by signing an executive decree. This allowed the Ministry of Health and the Costa Rican Social Security Fund to swiftly implement non-ordinary, bold country-wide measures to limit the spread of COVID-19, including public mobility restrictions and social distancing requirements.	Enforced through the Communicable Diseases Act of 2015 and multiple emergency decrees, the Thai Government similarly utilized a number of mandates, policies, and population-level public health and social measures to suppress COVID-19 transmission including public mobility restrictions, border control, masking, surveillance and contact tracing, and risk communication efforts. Thailand implemented a robust surveillance system that leveraged foundational infrastructure of past epidemics. Networks of surveillance teams, including the Surveillance and Rapid Response Team (SRRT) and Field Epidemiology Training Program (FETP), allowed for rapid deployment of health workers to support detection, isolation and treatment of active cases. At the sub-district level, the national network of Village Health Volunteers (VHVs) played a significant role in the COVID-19 surveillance, given their reach and familiarity with local communities. Laboratory and research capacity, which was significantly strengthened during the course of the pandemic, also contributed to Thailand's strong surveillance system.		In early 2020, the Sri Lankan government officially adopted a strategy to control COVID-19 that it described as the "Hammer and Dance" approach. At its core, the approach consisted of two elements: (i) effective border security to minimize entry of the virus, and (ii) aggressive actions to stop any new local outbreaks to bring transmission to manageable levels. Border security was ensured by restricting international arrivals, and eventually mandating 14-day quarantine and PCR testing for all arrivals. Actions to stop local outbreaks involved PCR testing to detect outbreaks, followed by contact tracing and isolation of identified cases and contacts. During the early months, over 2,800 Public Health Inspectors were deployed to support surveillance, contact training, and isolation efforts and provide food, medicines and hospital beds. Moreover, military assistance was utilized to build quarantine facilities, run community vaccination centers, conduct mobile vaccination and disinfection drives, impose curfews and travel bans. Mandatory masking and social distancing measures were also enforced.<br>• Actions to stop local outbreaks involved PCR testing to detect outbreaks, followed by intensive contact tracing and isolation of identified cases and contacts, and provincial, district and village level travel restrictions and lockdowns.	After Uganda's first case of COVID-19 on March 21, 2020, the National Task Force (NTF) turned its focus from preparedness to emergency response. It activated district task forces to coordinate subnational and local COVID-19 response activities like surveillance, contact tracing, and isolation. The NTF's pandemic response plan emphasized risk communications as well as community engagement to promote public health and social measures. The Incident Management Team managed Uganda's surveillance and laboratory systems and ensured logistics for supplies and transport, and deployed rapid response teams in local districts.	In Ghana, in order to enhance COVID-19 testing capacity and to maintain the integrity of samples from remote areas, drone technology was employed to shuttle COVID-19 test samples from rural areas to laboratories in Accra and Kumasi. Approximately 15,000 test samples were transported in a day and results were delivered via text message/SMS.
	**Strong partnerships with the private sector**	The CCSS established temporary partnerships to expand drug service delivery during the pandemic at times of more significant mobility restrictions to prevent patients from being left without treatment because they could not visit the health center. As a result, the delivery of medicines was carried out by the CCSS and under the cooperation of the national post office company, universities, and private companies such as rent a car. Then, the Proactive Testing Campaign, led by the Costa Rican-American Chamber of Commerce (AmCham), the CRUSA Foundation, and the Business Association for Development, raised more than US$1.8 million from 440 donors. The campaign delivered 10 PCR testing machines and 15,743 test kits according to the CCSS requirements. Also, public-private partnerships involving multisectoral collaboration between the Costa Rican Chamber of Commerce, Academia, and the Costa Rican Social Security Fund, significantly contributed to the procurement of necessary resources during the pandemic. The ALEPP initiative helped coordinate the Costa Rican value chain to produce necessary PPE in the country– they identified local companies, various production capacities, and critical inventory and raw materials. As a result, 600,000 face shields were manufactured locally by Grupo Vargas, which transformed its operations into plastic to adapt to the increasing PPE demand. The collaboration also identified protocols to decontaminate N95 masks using local companies' resources available in their production plants.	Public-private partnerships played a significant role in manging covid 19 across multiple domains.<br>For COVID 19 health utilization, private entities with the support of national covid 19 budget acted as the surge capacity both diagnostic (additional private laboratories) and treatment by providing additional access to both COVID 19 related inpatient and outpatient cares.<br>For vaccination coverage, private hospitals also acted as the vaccination sites in urban areas. Furthermore, multiple department stores in Bangkok also provided location to set up additional vaccination sites to increase vaccination coverage.<br><br>In an effort to also support the tourism sector that has been hard hit by the pandemic, several hotels were rented by the government to receive asymptomatic patients or mild cases in what are called "hospitels." Moreover, public-private sector collaborations also supported the stockpiling of PPE, medicines, and the installation of negative pressure isolation rooms and ICUs for hospitals in need.	The Dominican Republic leveraged past pandemic preparedness plans to develop their COVID-19 contingency plan, which laid the groundwork for strong PPPs in the country. Multisectoral coordination between the government and the private sector facilitated the procurement of hospital resources, strengthened testing capacity, and the roll out of an effective vaccination campaign. Leveraging the country's existing private laboratory system, the government was able to make all testing and treatment free-of-charge in all sectors during the first year of the pandemic. Furthermore, the Dominican health system successfully managed their COVID and non-COVID case load without exceeding the availability of hospital or ICU beds; this was attributed to an effective collaboration between the government and the private sector under the Health Cabinet leadership. Data on patient flow was centralized by the government and each day, the availability of hospital and ICU beds was modified by both the public and private sectors depending on capacity. Additionally, PPPs also contributed to the success of the country's vaccination campaign. Private companies helped finance vaccine purchases, manage storage and transport of vaccines, and provide physical space for vaccination centers. Private sector investment and support was credited with helping the Dominican Republic government procure vaccines early and vaccinate 77% of the population over 18 years of age with their first dose by May 2022.	• The private health sector provided paid services such as quarantine facilities, PCR testing and private ICCs for paying patients to avoid pressure on MOH, with set price ceilings. By December 2021, private laboratories undertook around 40% of all PCR testing, with some of them investing in high-throughput machines.	In order to support the overwhelmed government testing centers in Uganda, private sector laboratories were licensed and permitted to provide additional laboratory testing services. Partnerships with private companies, NGOs and foreign governments enabled the procurement of essential goods and PPE for health facilities and providers including CHWs.	In Ghana, a coalition of civil society, community-based organizations and private sector entities such as banks, pharmaceutical companies, and wealthy individuals, was activated to form an alliance that supported the procurement of PPE, test kits, and other resources needed to contain the pandemic. This made it possible to mobilize resources not just for health personnel but also for vulnerable communities and immunocompromised individuals during the early stage of the pandemic.
**National / Governmental / Population Level Measures**	**Existing financing mechanism(s) dedicated to supporting emergency response and EHS**	In 2016, the CCSS Board of Directors established a contingency fund (USD $200 million) to provide monetary resources in case of disasters such as earthquakes, floods, or fires that affect health services. The fund also received additional investments from the Central Government of Costa Rica and loans from international finance institutions. This contingency fund, only available to the public sector, was then expanded to include pandemics and became a fundamental factor in Costa Rica's preparedness. In 2020, the CCSS was able to swiftly respond to the COVID-19 pandemic leveraging the contingency fund which allowed for the increase in hospital capacity and infrastructure, adjustment of health personnel protocols, and procurement of necessary resources to maintain EHS. The contingency fund financed most of the resources and adaptation that the CCSS and public health system underwent throughout the pandemic. For instance, 36% of the fund was used to finance remunerations such as new healthcare jobs, paying for extra hours, and the accompanying social security costs. This includes adaptations like Costa Rica's Vacancy Substitution Scheme which supported the mobilization of health care workers from certain specialties to high-need COVID facilities. The second largest expenditure was on durable goods including purchasing new medical equipment (ventilators, ICU beds) for the COVID-19 specialized center (CEACO) and other hospitals, financing remodeling, and adapting the physical infrastructure of several hospitals across the country.	During the COVID-19 pandemic in Thailand, the central budget and emergency loan decrees were the two main sources of funding for public health emergency response. The central budget was allocated to the line ministries for overtime and risk compensation for public health related staff, vaccines, local quarantine, and state quarantine. The emergency loan decrees, which came from external and internal loans, were earmarked for the following purposes: (1) Risk compensation for public health related workforce, (2) Procurement of medical supplies, drugs, and vaccines, (3) Disease control, research, and vaccine development, (4) Treatment and quarantine, (5) Response plans for COVID-19 (e.g., cars for active screening, communication systems), (6) Additional funds were also made available to assist with other social support schemes to alleviate the economic challenges faced by the country during the pandemic. This includes social support for individuals across various sectors, including vulnerable populations such as the poor and informal sector workers.<br><br>Additionally, the Thai government facilitated rapid maneuvering of healthcare financing mechanisms to ensure that COVID-19 operations remained funded and funded separately from other non-COVID services, thus EHS have experienced minimal to no funding disruptions during the pandemic. These stable funding levels ensured many essential health services did not experience catastrophic disruptions during the pandemic. Without this continuity, essential services may not have been able to devote time and resources to adapting and innovating service delivery in the face of numerous challenges presented by the pandemic. This has been coupled with the activation of funds like the Community Health Fund to further support community-based health workforce networks (e.g. VHVs) to support both COVID and non-COVID efforts.	The Dominican Republic (DR) used a variety of strategies to finance their COVID-19 response and recover their economy. Most of the resources required to respond to the pandemic were financed by domestic sources. In 2021, the central government health allocation almost doubled that of 2019, with more than 60% of the funds allocated towards the vaccination program. Additionally, domestic public-private partnerships helped finance the Dominican Republic's resource procurement, especially vaccines. In collaboration with the private sector, the DR was able to acquire enough vaccines early in the process. However, there were also some external funds from international organizations, mainly as loans requested by the Government. The World Bank granted US $150 million to support the implementation of emergency measures to contain the spread of COVID-19 and manage the impact of the pandemic through the Catastrophe Deferred Drawdown Option. DR received additional assistance from the US Department of State, USAID, and the International Bank for Reconstruction and Development.		In the West-Nile region, the voucher system facilitated by Association of Volunteers in International Service (AVSI) Foundation and UNICEF with funding from Swedish International Development Agency (SIDA) under the District Health System Strengthening Project enabled the continuity of essential service delivery for mothers and newborns with the help of motorcycle riders who had been trained on guidelines for COVID-19 prevention and control of infections and given alcohol-based hand sanitizers and personal protective equipment, including face masks. Also, at the end of March 2020 Uganda's parliament approved a US$30.7 million (104 billion Ugandan shillings) budget for COVID-19 response, mobilizing financial resources from government and other international agencies to support this budget.	In response, the government constructed an infectious disease centre in Accra, to help contain Covid-19 cases. In addition, the government secured US$100 million start-up fund through the Ghana Investment Infrastructure Fund (GIIF) to construct 111 health facilities (Agenda 111); which will comprise 101 new 100-bed district hospitals, 7 new regional hospitals and 3 infectious diseases centres, with each facility expected to be completed within 12 months, starting from the point of commencement (MoH, 2020c).

Appendix Table 2. Country examples for all cross-cutting themes

Framework Category	Cross-cutting theme/practice	Costa Rica	Thailand	Dominican Republic	Sri Lanka	Uganda	Ghana
	**Government policies/initiatives in place to prioritize the maintenance of EHS**		In Thailand, the Department of Medical Services (DMS) under the MOPH established a national “New Normal Medical Services” initiative that was implemented across all levels of the health system with the ultimate goal of ensuring both COVID and non-COVID patients receive appropriate treatment and care. This consisted of a package of innovative approaches including digital health solutions to reduce crowding and control infections in healthcare settings while also promoting equity. Under the New Normal Medical Services and informed by pilot innovations, a comprehensive set of practices were designed and implemented across the country specifically focused on maintaining NCD services given that stroke and heart disease are leading causes of death in Thailand. The redesign of NCD services consisted of a five-step model that included (i) greater empowerment and engagement of patients including for self-monitoring, (ii) population management and clinical risk stratification to focus efforts on those that need them most, and (iii) a model of care that relied more on self-care and remote services. The NCD redesign also included multiple channels for home/community delivery of medications (e.g. by mail).<br><br>Moreover, in order to maintain immunization services during the pandemic, the Thai government employed a number of strategies including (i) creating separate well-baby clinics in hospitals to separate the sick from the healthy, (ii) introducing non-traditional vaccine venues (e.g., drive thru options, or vaccination at home), (iii) public communication efforts, and (iv) deploying catch up campaigns.	At the beginning of the COVID-19 pandemic, the Dominican Republic leveraged past pandemic preparedness plans to create and enforce their contingency plan, which emphasized both the importance of effective outbreak control and maintenance of essential health services. With technical support from Pan American Health Organization (PAHO), the Centers for Disease Control and Prevention (CDC), and the United States Agency for International Development (USAID), the Dominican Republic government was able to implement the contingency plan quickly and produce the “General Guidelines for the normalization of the provision of health services in the face of the COVID-19 pandemic”, in June 2020 which underscored the importance of maintaining EHS in the midst of a health emergency.	Similarly, the ‘Sri Lanka COVID-19 Preparedness and Response Plan’ published in April 2020 specifically recognized the maintenance of EHS as a priority during the pandemic. The public sector managed by the Ministry of Health ensured EHS continued to function throughout the country mainly through the release of government circulars targeted to relevant health organizations on a regular basis. For example, in March 2020, a circular was released instructing all government healthcare institutions to continue providing treatment for patients with NCDs while adhering to COVID protocols. Routine clinics providing EHS and emergency care and inpatient services for critical patients were to remain open. Sri Lanka also implemented policies that enabled multi-month drug dispensing, utilization of postal services to deliver medicines to patients, and private sector delivery of medicines. The Medical Officers of Health were responsible for ensuring all quarantined individuals were supplied with their required medication during the crisis.<br><br>Recognizing the potential for disruption, the Sri Lankan MOH also took proactive measures to ensure maintenance of maternal and child health (MCH) services. They specifically instructed all Medical Officers of Health to keep their clinics open, with suitable cover-up provisions made for midwives on sick leave. Procedures were modified as needed to keep MCH clinics open, and COVID safety protocols were introduced to allow home visits and clinic visits to continue. To minimize risk of transmission, MOH issued guidelines that prohibited private medical providers from managing suspected/confirmed pregnant women and newborns, and granted exclusive responsibility to the public sector to triage positive cases to designated national COVID-19 centers for management.	Even before Uganda reported a substantial number of COVID-19 cases, national officials acted swiftly to develop and implement key preventive measures such as movement restrictions and mask mandates. in May 2021, Uganda re-implemented a series of travel restrictions to reduce the risk of importing new variants. (For instance, no travelers or flights from India were allowed into the country.) They also halted the phased re-opening of schools and institutions of learning, restricting community gatherings, limiting the capacity and hours of operation of public transport, longer curfews, and strict mask wearing and hand hygiene in public places. On June 6, 2021, officials implemented a partial lockdown; on June 18, this became a complete lockdown. (Let's omit this example)<br><br>Following the implementation of NPIs which disrupted maintenance of essential health services, the Ministry of Health established a committee on maintenance of EHS at both national and subnational levels to guide continuity of services. The committee also developed guidelines on maintenance of EHS that were disseminated to the various levels of the health system. Districts and other stakeholders were invited to present to the national committee and where disruptions were noted, interventions were proposed and implemented. At subnational level- regional, district and facility levels, the government established sub-committees to provide oversight and coordinate the maintenance of essential health services.	To expand and broaden testing coverage, drones were deployed in the delivery of medical supplies and biological samples collected from suspected coronavirus patients in remote areas to the only two major laboratories with the capacity to perform covid-19 testing in Kumasi and Accra, allowing the Ghanaian health system to more quickly monitor and appropriately/efficiently respond to changes in the transmission of COVID-19 virus. During the roll-out of vaccines and vaccination campaigns, drone technology was also deployed to deliver Covid-19 vaccines to remote areas, thus enhancing the vaccination campaign and increasing vaccination coverage.<br><br>Another innovative strategy employed by the frontline health workers in the health facilities was the adoption of the appointment system, which saw a reduction of the effects of the inadequate number of frontline health workers in the facilities due to additional tasks and responsibilities resulting from COVID-19-related activities. The appointment system involves the assignment of patients that were not severely ill to days of the week when the health workers were less busy.<br><br>An additional innovative strategy was adopted to relocate routine essential health services, which required simpler procedures; such as ANC, routine immunization, etc., from health facilities to homes where these were conducted in safer environments via the home visit approach by the health workers; thus ensuring that patients were not denied EHS
	**Expansion of UHC and/or other health financing schemes to increase care coverage**	The Board of Directors of the Costa Rican Social Security Fund approved the extension of health insurance coverage to workers who are under a labor contract suspension from the beginning of the pandemic through June 30, 2021, to support households with reduced economic income due to pandemic-related unemployment.	Thailand coupled existing UHC schemes with newly introduced health financing mechanisms in order to provide full coverage of quality COVID-19 services without co-payment. For instance, the government expanded health insurance coverage for migrant workers. While migrants working in the formal sector are covered under the SSS, with equal access to social security benefits and health services as Thai citizens, those who work in the informal sector are covered through the Health Insurance Card Scheme, which is open to all documented and undocumented migrants and their dependents. Financial protection of underserved/low-income communities ensured access to COVID-19 care and essential health services and supported their overall health and wellbeing during the pandemic.	In the Dominican Republic, as a result of a policy adopted by the government to prioritize the financial protection of its people, the Family Health Insurance (SFS) increased coverage from 77% of the population before the pandemic to 96% in December 2020 and 98% in May 2022.	Support measures for households primarily consisted of cash grants to households. These included one-off or monthly grants of Rs 5,000 (USD 27) to low-income earners or the elderly, and COVID-19 or lockdown affected households during April–December 2020; Rs 10,000 (USD 55) interest free advances to welfare (Samurdhi) recipients; and Rs 15,000 (USD 82) worth of dry rations to families regardless of economic status who were under quarantine for two weeks. Other assistance included giving one month grace periods to the public for paying utility and credit card bills less than Rs 50,000, and increases in insurance benefits for healthcare workers, police, the military and other government employees.		Ghana has a National Health Insurance scheme that was established in 2003 and covers about 40% of the population. There are also other private health insurance providers bringing the total percentage of the population insured at 70% according to the 2021 national census.
	**Strong academic-government partnerships facilitated data-driven decision-making**	Researchers in Costa Rica from universities such as the University of Costa Rica, INCAE Business School, and Hispanoamerican University were actively involved in COVID-related data analyses that were presented weekly to the President of the Republic. These analyses were reviewed as part of the multi-institutional decision-making process for pandemic response within the National Situation Analysis Room of the Emergency Operations Center.	Thailand’s robust academic and research networks, both connected to and independent of the Government, have been highly active during the pandemic and have been able to document and facilitate data-driven decision-making at all levels. This highlights the strong connections between these research entities and health decision-making bodies, which have been able to work together to regularly analyze COVID-19 surveillance data, inform policymakers and provide guidance on prevention and control measures.	In the Dominican Republic, the Technological Institute of Santo Domingo (INTEC) developed a predictive model for the epidemiological behavior of COVID-19 to manage data on cases, mortality, non-pharmacological interventions, and health planning in order to monitor the pandemic. The university also supported case detection in the early days of the pandemic, offering software that worked as a fast diagnostic test, asking key questions to infer the possibility of infection, and providing recommendations. This model was used by the Direction of Epidemiology to help with case management and reduce the burden of hospitalizations.	University laboratories analysed and released information about the distribution COVID-19 cases variants which were sometimes used as the basis for implementing control measures such as isolation, quarantine, and lockdowns.<br>Academic expertise was used in several government COVID-19 related technical advisory groups on public health response, control strategies, vaccine evaluation, regulation and vaccine deployment strategies.	The Ugandan National Task Force established a scientific advisory committee made up of interdisciplinary public health specialists and academicians from the Makerere University’s schools of public health, medicine and statistics; the Medical Research Council; and the Uganda Virus Research Institute to collate, synthesize and interpret emerging data and translate new and evolving information into evidence-based policies and strategies for pandemic response.	During the survey, it was noted that decisions to implement social and non-pharmaceutical interventions were primarily driven by data and science; a strategy, which contributed to the success story of COVID-19 management. It was revealed that the Ghana Health Service (GHS) sought the support of external experts and reliance on in-country infectious disease modelers who developed standard mathematical models to assess the effectiveness of different interventions; including forecasting or predicting the epidemic scenarios contributed significantly in terms of where scarce resources were directed to achieve the greatest impact.
	**Robust vaccination efforts**	The CCSS formed alliances with private organizations such as soccer teams, shopping malls, and the media to massify access to vaccination; for rural areas, CCSS allowed the directors of the health areas to allocate the necessary resources to carry out vaccination campaigns using the ATAPS in remote locations in rural areas. By December 31, 2021, the CCSS vaccinated 77% of the population, and 69% of the population were fully vaccinated, mostly with Pfizer and Astra Zeneca.		The DR Ministry of Public Health leveraged existing structures responsible for administering immunizations for vaccine-preventable diseases such as the Expanded Program on Immunizations (PAI) to provide the inoculations. A digital platform (“Get Vaccinated DR”) was launched to provide COVID-19 vaccine related information to the public. Moreover, private companies and small businesses also encouraged their employees to get vaccinated with the goal of re-opening the economy.	The Sri Lankan NCC developed a detailed ‘National Deployment and Vaccination Plan for COVID-19 Vaccines’ in January 2021 outlining all aspects of COVID-19 vaccination from planning, regulatory, costing, prioritization, service delivery, logistics, human resources, advocacy, safety and data management. Vaccinating the adult population with COVID-19 vaccines became a key policy goal by early 2021. In addition to vaccination centers at public, private and military-run health facilities, mobile vaccination drives were launched targeting individuals who were unable to travel to vaccination centers, including elderly people and old age homes as well as large organizations like garment factories and IT companies		
	**Service delivery adaptations and provision of care through alternative modalities to minimize disruption of EHS**	The first element that had enormous weight in maintaining EHS during COVID-19 was improving coordination between health centers through an integrated network management strategy. This strategy, also known as “One CAJA,” improved the coordination of the main healthcare provider in Costa Rica, the CCSS, which worked as an integrated unit throughout all its different levels of care and health facilities. The One CAJA included protocols and preparation of physical spaces, beds, workforce, and equipment to provide health services separately to patients with COVID-19-related symptoms and other patients with non-COVID-19-associated pathologies. One CAJA’s main objective was to avoid further contagion in patients attending non-COVID-19 connected health care by limiting the contact of COVID-19 patients with the resources assigned to the rest of the system. In March 2020, anticipating the high demand for hospitalization services and ICU beds, Costa Rica repurposed rehab centers such as the “Centro Especializado de Atención de Pacientes con COVID-19” (CEACO) into hospitals dedicated to caring for COVID-19 patients.	Thailand employed a number of service delivery adaptations to maintain access to essential health services. A primary way NCD services were maintained was through the ‘New Normal Medical Services’ Initiative, implemented to ensure both COVID and non-COVID patients receive appropriate treatment and care. NCD redesign was a key component in the initiative, promoting digital innovations, such as remote self-monitoring, patient risk stratification and alternative medication dispensing. The model leveraged existing infrastructure to make efficient use of limited resources, focusing on patient-centered services, with care devolved to the community level. This was done through mobilizing existing Village Health Volunteer (VHV) networks, on the background of years of investment into primary care systems and health personnel recruitment.	Since big hospitals tend to attract the most patients, during 2020 and 2021, the SNS put a strategy in place to ensure they did not reach max capacity. An agreement was made with the 911 emergency unit to ensure ambulances in selected hospitals. With the data on occupancy by hospitals, the SNS sent the patients that were not in need of the exclusive treatment offered by these hospitals to less visited and occupied health centers depending on the complexity and severity of the case (interviews, policy makers, frontline respondents).	In collaboration with the private sector, the Sri Lankan government established Intermediate Care Centers (ICCs) in hotels across the country serving patients with mild to moderate COVID-19 symptoms in order to reduce the burden on public facilities. The military also built quarantine centers in key areas with MOH support	Initially, health officials in Uganda isolated all confirmed COVID-19 patients at designated health care facilities. As cases increased, secondary regional isolation centers (such as the Mandela National Stadium in Namboole) were used to manage non-severe, high-risk patients, typically those with comorbidities. Subsequently, officials implemented home-based isolation nationwide for asymptomatic people who tested positive for COVID-19 and those with mild symptoms and no known risk or comorbidity. Medical teams instituted by district task forces contacted isolated patients daily to monitor their symptoms, and patients could also contact the medical surveillance team using a national toll-free hotline. Furthermore, officials designated 17 regional referral hospitals as COVID-19 treatment facilities and installed intensive care unit beds. To avoid further disruption to EHS services, some countries found unconventional ways to make sure patients were provided care, with Uganda using motorbike drivers to get women to maternal health clinics.	To enhance COVID-19 testing capacity and to maintain the integrity of sample from remote, rural areas, drone technology was employed to shuttle COVID-19 test samples from rural areas to laboratories in Accra and Kumasi. Approximately 15,000 test samples were transported in a day and results were delivered via text message/SMS. An additional innovative strategy was adopted to relocate routine essential health services, which required simpler procedures; such as ANC, routine immunization, etc., from health facilities to homes where these were conducted in safer environments via the home visit approach by the health workers; thus ensuring that patients were not denied EHS.
	**Rapid mobilization and deployment of health workforce for COVID response and EHS delivery**	The CCSS managed almost 58,000 health care workers within its network of health facilities. The pandemic showed that to maintain EHS and effectively address the fight against COVID-19, it was necessary to hire additional temporary personnel of all types (e.g., physicians, nurses, administrators, etc). The access to some types of specialist physicians, such as internal medicine and intensive care doctors, was limited, denoting the need—for the country and the CCSS—to invest in developing certain specialists. It also became apparent the need to have more flexibility in the job descriptions of health care workers’ contracts to deploy this resource in relevant tasks within their capabilities during an emergency.	In early 2020, while cases had been recorded in 68 out of the 77 provinces, the top ten provinces accounted for almost nine out of 10 of the total cases (87%). This prompted MOPH to mobilize surplus capacity from other provinces, as a shortage of specialists, in particular intensive care nurses, critical care experts, and epidemiologists, became evident in certain locations. Additionally, some hospitals deployed experienced nurses from non-intensive care units within their own hospital or province to support on-the-job training for the intensive care unit and infection prevention and control (IPC). Moreover, short-course tutorials were provided to nurses on acute respiratory distress syndrome. In provinces with a high caseload and critical shortage of healthcare workers, medical teams were mobilized from other provinces, with MOPH closely monitoring at a provincial level. Mobilizing existing Village Health Volunteer (VHV) networks, on the background of years of investment into primary care systems and health personnel recruitment.	One of the strengths of the response to the pandemic has to do with health personnel. Many of the key informants confirmed their willingness to serve, one of them said “they were willing to face anything, even to give their lives to be there during the pandemic and working on the front lines. There was never an exodus or strikes, as happened in other countries”. As noted, more than 3,000 physicians and other health workers were hired, and significant incentive bonuses were awarded to front-line staff.	Sri Lanka remobilized field staff from other health programs (eg, mosquito-borne disease control) and medical officers from non-clinical areas to help manage the outbreak allowing EHS staff to continue providing EHS.	In some districts in Uganda, staff were recruited and redeployed to maintain essential health services, while others were assigned to provide care at COVID-19 facilities. For example, 250 health workers—including epidemiologists, doctors, anesthetists, nurses, laboratory technologists, psychiatric clinical officers, ambulance assistants, and drivers—were recruited on contract for 6 months and deployed to the COVID-19 treatment centers and to support districts and Points of Entry in surveillance. Also, facilities designation deployed some staff to provide COVID-19 services, keeping the rest at facilities providing non–COVID-19 services. Community health workers, medical and public health students contributed to efforts of contact tracing and provision of essential health services. Village Health Teams were mobilized and trained to serve their communities.	The lower-level staff of the Ghanaian health system was given additional training on how to handle covid-19 patients without undue cross-infection by focusing on strengthening existing health structures, logistics, and human resource capacity in preparedness and readiness for any worsening of the outcomes. Most facilities relied on existing emergency plans that showed how infectious diseases, in general, should be handled once they record the first case at the facility. In addition, the existing preparedness plans for emergencies were updated to contextualize the situation prevailing under the current COVID-19 disease. Most of the frontline health workers felt safe with the updated emergency plan that was put in place.

Appendix Table 2. Country examples for all cross-cutting themes

Framework Category	Cross-cutting theme/practice	Costa Rica	Thailand	Dominican Republic	Sri Lanka	Uganda	Ghana
**Measures for Service Delivery & Workforce Adaptations**	**Digital health solutions**	The order to reconvert services was crucial for increasing flexibility and innovation in the middle of outbreaks as it allowed the massification of practices already being adopted on a small scale in CR; for example, some alternative modalities such as telemedicine and telemonitoring, and auxiliary services as home delivery of medicine were enhanced during the emergency as a solution to the need to maintain COVID-19 care services and active EHS.	For both COVID-19 and EHS services, the provision of care through alternative modalities, including telemedicine and telemonitoring have been utilized during the pandemic. For example, underneath Thailand's NCD redesign initiative, telehealth services are aligned with patient risk stratification groups to limit the number of in-person visits and more efficient use of patient-centered primary care. This is also coupled with the expanded adoption of different medication delivery options (home delivery, etc) and the utilization of the village health volunteer (VHV) network in many communities. Further studies are required to better ascertain the full scope and scale of these new modalities and whether they have been equitably utilized.	Regarding Telemedicine in DR, in November 2020, the National Health Service (SNS) and the Emergency and Health Management Committee to combat COVID-19 launched the Critical Care Telemedicine Project, with the aim of interconnecting health personnel of provincial hospitals with high-complexity base centers specializing in critical care. The country has some private Telemedicine platforms such as Hulipractice, which is a remote consultation tool, free for 3 months, with video consultations, a digital agenda to schedule your appointments via WhatsApp, Facebook or website; electronic prescriptions and digital clinical history. Also, some private clinics such as the IMG Hospital, in Punta Cana, have the service of treating patients remotely and scheduling their appointment. As of the pandemic, private ARS such as ARS Humano, introduced the telemedicine service with the company TELEMED, which provides audio or video conference services with a network of doctors who provide information and guidance, interpretation of test results and diagnosis, as well as electronic prescriptions.	Through partnerships with the private sector, Sri Lanka's leading telecommunications provider Dialog donated mobile phones and broadband routers to government hospitals and quarantine centers to coordinate patient treatment. Primary care providers in the public sector were given detailed guidelines on implementing telehealth services within their respective clinics. This included following a remote consultation algorithm to differentiate suspected COVID-19 patients from non-COVID and managing them accordingly. Concurrently, medical schools and hospitals such as the University of Kelaniya published phone numbers on their department websites, and patients were given information on how to seek medical advice through tele-consultation. Patients also used WhatsApp to send photos of symptoms or lesions, and doctors used SMS to send prescriptions to patients. Video consultation services were also made available through WhatsApp by the majority of consulting doctors at private hospitals, often through existing appointment booking systems online. Teleconsultations were mainly used for post-surgical follow-up such as cesarean sections, elective surgeries or other emergencies, and are still ongoing in 2022.	The COVID-19 pandemic pushed healthcare providers to use digital technologies to remotely deliver clinical and non-clinical services: for instance, stakeholders such as the medical concierge group, Infectious Diseases Institute (IDI) and Baylor Uganda adopted telehealth services and supported several cross-cutting areas including HIV/AIDS, tuberculosis, maternal newborn and child health, sexual and reproductive health services. These telemedicine applications helped maintain access to care during the early months of the COVID-19 pandemic in Uganda, from teleconsultations with care providers (including telepsychiatry and tele-pharmacy services) to the dissemination of health information via mobile phones	Some facilities employed innovative ways of providing health services to their clients. One of such innovative approaches was the use of telemedicine."We had an app, COVID Connect, by which people we treated and discharged could keep in contact with us, and people with symptoms could go into the app and indicate their symptoms for some healthcare professionals from our facility to contact them." (IDI11—Quaternary facility, Greater Accra Region)<br><br>During the roll-out of vaccines and vaccination campaigns, drone technology was also deployed to deliver Covid-19 vaccines to remote areas, thus enhancing the vaccination campaign and increasing vaccination coverage.
	**Operational flexibility to transfer patients and resources between facilities**	Due to its highly integrated healthcare system and proactive health system leadership, Costa Rica had the distinct opportunity for a coordinated pandemic response plan. The Costa Rican Health System (NHS) is a vertically and horizontally integrated health system, in which the Costa Rican Social Security Fund (CCSS) oversees and manages all levels of public sector healthcare provision (over 90% of the population). The fact that a single institution, the CCSS, controls a whole network of public clinics, hospitals, and centers located throughout the country allowed the CCSS, private partners, and health system leadership to respond quickly to the regional surges of COVID-19 across all levels and sectors. This multilevel strategy comprises of an Operational Coordination Council in which the directors, administrators, managers, and heads of health facilities within the public health sector meet monthly to present the specific needs of each public health facility and coordinate resources, patient movements, and priority specialties of care according to the demand for services. During the pandemic, the Operational Coordination Council met weekly and was essential to real-time health system capacity decisions and country-wide patient transfers. The digital health platform "Expediente Digital Único en Salud" (EDUS) was an essential tool that provided government decision makers with real-time data on the health system such as monitoring bed occupancy rate by health facility, managing patient transfers and ensuring hospitals do not reach max capacity.	Advanced cases requiring specialized care are then referred or transferred towards the provincial level hospital, commonly known as a general hospital. In very advanced cases, if the use of multi-disciplinary care provides better outcomes for the patient, the patient may be then referred towards regional hospitals or academic excellence centers.	In April 2020, the Dominican government integrated the Command, Control, Communications, Computers Cybersecurity and Intelligence Center of the Ministry of Defense (C5i) with the health system, specifically the National Health Service (SNS) and the Ministry of Health (MOH). They developed a digital platform that centralized data from hospitals, clinics, laboratories, pharmacies and insurance converges, and presented in real time the number of beds available and in use in a hospital, the Intensive Care Units (ICUs), the ventilators, the ambulances as well as other data that contributes to creating predictive models to be able to make the best decisions and predictions on the resources that are required. Through partnerships with the private sector, the government was able to procure the necessary resources and avoid hospital overload. The platform was most useful in ensuring that large hospitals did not reach max capacity. With the data on occupancy by hospitals, the SNS sent the patients that were not in need of exclusive treatment offered by these hospitals to less occupied health centers depending on the complexity and severity of their cases. There was also an artificial intelligence (AI) system that allows, through data analysis, to make projections and develop epidemiological profiles in order to predict the behavior of the virus and act accordingly.	Initially, all confirmed cases were isolated in hospitals. But later to reduce pressure on the hospital system and to prioritize attention to severe cases, management of stable and asymptomatic COVID cases was moved out of public hospitals and into purpose-built Intermediate Care Centers (ICCs). Cases were then managed in three levels depending on their severity: Level 1- Intermediate Care Centers; Level 2- Selected COVID-19 Hospitals; Level 3- COVID-19 designated specialized/tertiary care hospitals.<br>Transportation of new cases was managed by the ambulance services, and other transfers between facilities were undertaken by the military.		
	**Adaptation of existing health infrastructure and disease response capacities**	In addition to the CCSS Contingency Fund, the Head of the Health Economics Unit of the MoH mentioned that US $ 40 million from different MoH programs were temporarily assigned to the COVID-19 emergency. For instance, AIDS and Vector-borne diseases budgets from 2020 and 2021 were partly transferred to the COVID-19 crisis (in 2022, MoH reassigned budgets to the affected programs)	Thailand has had a strong track record in controlling disease spread (SARS in 2003, H1N1 in 2009, and MERS in 2016). Established in 2004, over 1,000 Surveillance and Rapid Response Teams (SRRT), composed of public health nurses and officers, have been positioned across the country to rapidly detect and respond to emerging public health threats. During the COVID-19 pandemic, many of these teams were deployed to rapidly isolate cases, provide treatment, and actively trace and quarantine contacts. Stationed at provincial health offices and district hospitals (and networked with sub-district health centers), SRRTs typically comprise public health nurses and officers. Additionally, the long established national Field Epidemiology Training Program has trained thousands of experts in disease outbreak investigation and control, many of whom were stationed at the provincial and district level to manage COVID-19 outbreaks and conduct contact tracing and epidemiological data analyses. At the sub-district level, the national network of Village Health Volunteers (VHVs) played a significant role in the COVID-19 surveillance and community engagement, given their reach and familiarity with local communities. Ultimately, all these existing disease response networks working in concert created a robust surveillance system that enabled a strong COVID-19 response in Thailand.	Due to these past experiences, response plans were developed in three phases: pre-outbreak monitoring and community outreach with prevention, during epidemic prevention strategies and health system response, and post-epidemic reporting and lessons learned. The DR created multiple outbreak preparedness and response plans including one for chikungunya, the first of its kind in the Americas, and one for the influenza epidemic. Additionally, they established a national communication plan, with radio and television messages in simple language addressing prevention, posters, and booklets for schools with guidance for the educational community and the general public. The National Council for Prevention, Mitigation and Response to Disasters (CN-PMR), created in 2002, is the governing body on disaster preparedness and oversees guiding, directing, planning and coordinating the national system. The law orders the preparation of a Contingency Plan with specific pre-established procedures for coordination, alert, mobilization, and response to the occurrence or imminence of a particular event, such as an epidemic or pandemic.	Existence of strong health system with experience and competency in disease surveillance, contract tracing, and handling of infectious disease outbreaks; a technically competent, efficient national immunization program; a system of hierarchical control and management of public health services that could facilitate rapid, uniform response across the whole island; and close alignment at the district management level of its preventive health services with the public sector's curative side.<br>Universal healthcare and good access to hospital services country-wide<br>Ambulance service<br>Testing was expanded to a network of 27 laboratories mostly through repurposing of existing laboratories, supported by establishment of new laboratories. Existing private hospital laboratories modified their facilities to commence COVID-19 PCR testing.	Uganda deployed more than 10,000 CHWs (locally referred to as Village Health Teams established during previous epidemics) trained on infection prevention and control, epidemic surveillance, and other aspects of outbreak response. The country repurposed their outbreak coordination and laboratory capacity built for other diseases, such as HIV and tuberculosis, for COVID-19 testing. Existing laboratory transport networks facilitated the rapid scale-up of diagnostic testing capacity, along with systems for quality assurance.	Prior to the confirmation of the Covid-19 outbreak in Ghana, the national disease surveillance department of the GHS developed a response strategy to repurpose the nation's ILI surveillance system to trace and track infected persons and high-risk individuals and treat all confirmed cases
**Community / Patient Level Measures**	**Mental health and psychosocial support programs for the community**	The Ministry of Health, the 9-1-1 Emergency System, and the College of Psychology Professionals of Costa Rica are implementing a joint project to provide psychological support to people affected by the changes in the environment due to the national emergency caused by COVID-19. The personnel was volunteer psychologists with extensive experience in crisis intervention. The "Psychological Support Office" could be contacted through the 9-1-1 line, 24 hours a day, and it was exclusive to assist people affected by situations related to COVID-19 so that other psychologies could attend to issues such as child protection and domestic violence.		The contact tracing application or "contact tracing" of COVIDRD as a prevention and follow-up measure, since July 15, 2020 (MSP, 2020). This application allows, among other functions such as if the user is a COVID-19 positive person, receive notification of the "epidemiological discharge" and the "serological discharge" as well as follow-up on their symptoms and mental health advice to help them overcome the disease.	Sri Lanka prioritized promoting mental health and psychosocial wellbeing early in the pandemic by extending the National Mental Health Helpline to all districts. The Directorate of Mental Health of the MoH (DMH), the Sri Lanka College of Psychiatrists, the WHO and the Mental Health and Psychosocial Support (MHPSS) provided continued access to essential mental health services and medications throughout the pandemic. Guidelines for health administrators to promote the mental wellbeing of frontline health workers and curtail future mental health conditions were also developed by the DMH.<br>When concerns arose about escalating domestic violence during the lockdown, MOH issued guidelines for hospitals to open "Mithuru Piyasa" - a friendly haven for survivors of gender based violence, which operated during usual working hours of hospitals.	Uganda focused on children's mental health. The NGO Save the Children provided psychosocial support through regular phone calls, home visits, mini gamebooks and radio messages to reduce stress among children. Additionally, the Buntu bulamu, a public project which aims to improve participation, inclusion, and quality of life for children with disabilities in Central Uganda, implemented a peer-to-peer support intervention for those children, along with their non-disabled peers, parents, and teachers. Across the country, CHWs were trained and encouraged to provide psychosocial support to clients.	Ghana provided support programs for healthcare workers' wellbeing.
	**Robust community engagement efforts leveraging CHW networks**		Thailand implemented promising service delivery practices to reduce fear/stigma and maintain utilization of essential health services. Among others, these include the implementation of the "New Normal Medical Services" frame to reorganize and redesign health center settings, NCD redesign efforts which utilizes self-monitoring capabilities with digital infrastructure, and adaptations to medication delivery to more efficiently allocate resources across the health system, and the mobilization of the extensive VHV network alongside primary care units to support meaningful community engagement and care.		Sri Lanka implemented an effective risk communication program using social media (Youtube, Facebook, Twitter), displaying of physical materials (banners, posters) and awareness activities led by relevant health representatives from the MOH. Communications focused on the importance of social distancing, public safety and respiratory etiquette, non-discrimination and anti-stigma, and psychosocial support. In addition, a mobile unit was sent out to the most vulnerable districts which screened awareness videos at public spaces, places of worship, government venues and schools.	The MoH developed health messages informing the public about the continued availability of other services in lower-level health facilities and regional referral hospitals, in addition to the COVID-19 treatment and management. The ministry also supported self management strategies for chronic diseases and the delivery of service within communities such as through outreaches.	Ghana reduced stigma and anxiety associated with COVID-19 infections in similar ways, focusing on risk communication and general health education to counter the mis/disinformation in the communities.
